# The experiences of consumers, clinicians and support persons involved in the safety planning intervention for suicide prevention: a qualitative systematic review and meta-synthesis

**DOI:** 10.3389/fpsyt.2024.1482924

**Published:** 2024-12-20

**Authors:** Edward O’Connor, Kate Rhodes, Nicholas Procter, Mark Loughhead, Alexandra Procter, Julie-Anne Reilly, Sophie Pettit, Monika Ferguson

**Affiliations:** ^1^ Mental Health and Suicide Prevention Research and Education Group, Clinical and Health Sciences, University of South Australia, Adelaide, SA, Australia; ^2^ Health & Biosecurity, Commonwealth Scientific and Industrial Research Organisation, Adelaide, SA, Australia; ^3^ School of Public Health, The University of Adelaide, Adelaide, SA, Australia; ^4^ Mental Health Short Stay Unit, Royal Adelaide Hospital, Central Adelaide Local Health Network, Adelaide, SA, Australia

**Keywords:** suicide prevention, lived experience, safety planning intervention, qualitative review, meta-synthesis

## Abstract

**Background:**

The Safety Planning Intervention (SPI) is an efficacious brief intervention for supporting people experiencing suicidal ideation and behavior. However, the subjective experiences of those who have used the SPI have not been systematically evaluated. This systematic review synthesized qualitative evidence regarding the experiences of people involved in the SPI.

**Method:**

Systematic searches of international, peer-reviewed, English language literature were conducted in seven databases (CINAHL, Embase, Emcare, MEDLINE, PsycInfo, Scopus and Web of Science).

**Results:**

A total of 588 articles were screened for eligibility, with screening, data extraction, and critical appraisal conducted in duplicate. Qualitative data were extracted from 10 included studies and synthesized via meta-aggregation. Ninety individual findings were aggregated into 14 unique categories, with categories subsequently combined to produce four synthesized findings: acceptability and positive outcomes associated with the SPI; maximizing the effectiveness of the SPI; navigating the involvement of support persons in the SPI process; barriers and limitations associated with the SPI.

**Discussion:**

Collectively, findings indicate that the SPI is viewed as beneficial by users and can be enhanced through clinicians’ use of a person-centered, collaborative approach, as well as through the inclusion of support persons. Future research should seek lived experience understandings from more diverse stakeholders, particularly regarding consumers’ experiences of using the SPI during acute distress. Further research is required to investigate causal pathways between SPI engagement and improved outcomes.

**Systematic review registration:**

https://www.crd.york.ac.uk/prospero/display_record.php?ID=CRD42022312425, identifier CRD42022312425.

## 1 Introduction

Suicide is a global health issue, with over 700,000 people dying by suicide each year (1). In Australia, approximately nine people are lost to suicide each day (2). Recent estimates suggest that for each death by suicide 135 people are exposed (3), indicating the wide-reaching impact of suicide and the potential for further distress for individuals, families and communities. In addition to suicide deaths, one in six Australians aged 16-85 years have experienced suicidal thoughts or behaviours in their lifetime (4).

Suicide prevention interventions can reduce suicide deaths and behaviors (5), and numerous brief interventions exist to support people experiencing suicide-related distress (6). One intervention that has been gaining popularity in both clinical and community settings is the Safety Planning Intervention (SPI; 7). The SPI involves developing a personalised list of coping and personal support strategies for use during the onset or worsening of suicide-related distress, typically through six components: a) recognising individual warning signs for an impending suicidal crisis; b) identifying and employing internal coping strategies; c) using social supports to distract from suicidal thoughts; d) contacting trusted family or friends to help address the crisis; e) contacting specific mental health services; f) eliminating or mitigating use of lethal means (7). Although widely used with US military veterans, the flexibility of the SPI has been demonstrated through its application across diverse age groups (8, 9), settings (10), and with varied populations including refugees (11), autistic people (12) and individuals recently incarcerated (13). The SPI has also been incorporated within or alongside wider therapeutic approaches, such as motivational interviewing (14). Traditionally completed in hard-copy format, the SPI has more recently been adapted to various digital versions (e.g., 15,16) which can be used in clinical settings or accessed by the public without clinical support.

Two recent systematic reviews (17, 18) and one meta-analysis (19) have explored the effectiveness of the SPI and safety planning type interventions. Through narrative synthesis of results, two of the reviews (n = 20 studies, 17; n = 22 studies, 18) concluded that this intervention contributes to reductions in suicidal ideation and behaviour, as well as suicide-related outcomes, such as depression and hopelessness, and improvements in service use and treatment outcomes. While the meta-analysis of six safety planning type studies (19) also found reduced suicidal behaviour among intervention participants compared to treatment as usual, this study found no evidence for effectiveness on suicidal ideation. Thus, despite the difference in findings related to ideation, current evidence generally supports the efficacy of the SPI in improving people’s coping capacities and safety, with benefits particularly pronounced for reductions in suicidal behavior. However, less emphasis has been dedicated to understanding the underlying processes by which people using the SPI derive benefits (20). While there is emerging evidence linking the quality and personalisation of safety plans to reduced suicidal behaviour and hospitalisations (16, 21), these mechanisms have been quantitatively assessed, rather than qualitatively described from the perspective of those who have used a safety plan. Contemporary thinking recognizes the critical role that lived and living experience plays in suicide prevention research yet there has been limited integration of lived experience in the development of existing suicide prevention interventions (22). Incorporating lived and living experience understandings into all stages of suicide prevention research is essential for ensuring that suicide prevention strategies meet the needs of those they have been designed for. Moreover, a personalized understanding of peoples’ experiences of using the SPI is needed to inform clinical practice, policy, and future research to enhance the effectiveness of the SPI and ultimately reduce the incidence of suicide and suicide-related distress.

### 1.1 Aims

This review aims to complement quantitative reviews and meta-analysis (17–19) by synthesizing the existing qualitative, peer-reviewed evidence regarding the experiences of diverse stakeholders (consumers, support persons, and clinicians) involved in the SPI. These stakeholder experiences include but are not limited to: what is perceived as helpful and unhelpful about safety planning; what processes facilitate positive effects; the collaborative process regarding how the safety plan is developed, used, accessed, and revised; as well as the perceived impact of the safety plan on suicide-related outcomes and other well-being indicators.

## 2 Method

This systematic review followed the PRISMA 2020 guidelines (23) and was conducted according to the Joanna Briggs Institute (JBI) methodology for systematic reviews of qualitative evidence (24). The review protocol was pre-registered with PROSPERO (CRD42022312425).

### 2.1 Search strategy and information sources

The search strategy was developed by MF, based on a previous safety planning systematic review (17), and refined in consultation with an academic librarian. We conducted searches on 28 November 2023 in seven databases: Embase, Emcare, MEDLINE and PsycInfo, in the Ovid platform; as well as CINAHL, Scopus and Web of Science. The final search strategy was broad, including terms for safety planning and suicide. Additional terms were trialed (e.g., for participant groups and study designs), however these restricted results and were excluded from the final strategy. We limited results to English language and a publication date range of 2000 to present. See **Supplementary Data Sheet 1** for the search strategies used in each database. Reference lists of included articles were pearled in duplicate (MF, EO, KR) for potentially relevant studies.

### 2.2 Study selection

Search results were imported to EndNote 21 (Clarivate, Philadelphia, USA) to manually identify and remove duplicates (MF). We screened the remaining results using Covidence (Veritas Health Innovation, Melbourne, Australia) in two stages, in duplicate: 1) title and abstract screening (MF, KR); 2) full-text screening (MF, EO, KR). Reviewers discussed any disagreements until 100% consensus was reached.

### 2.3 Eligibility criteria

Eligibility criteria included: published in English language; qualitative in design (or mixed-methods, but where qualitative data were able to be extracted); participants of any age who had direct involvement in safety planning (including consumers, support persons, service providers, clinicians, etc.) in any setting (e.g. emergency department, inpatient, outpatient, community, online, school, etc.); and where it was clear that safety planning was based on the Stanley and Brown (7) version. Studies could include the SPI as a standalone intervention, or as part of a wider intervention approach. Studies were excluded if they: were not published in English; were not primary research; were not qualitative in design (either purely quantitative or where qualitative method and data could not be extracted); participants had no direct involvement in safety planning; or where the type of safety planning intervention was irrelevant or unclear (i.e., no reference to Stanley and Brown, and/or no definition or description of safety planning procedures).

### 2.4 Data extraction

We custom-built an electronic survey (LimeSurvey, Hamburg, Germany) to extract key information from the included studies, including: aim; study location and setting; study design; participant characteristics (sample size, population description, age, sex); SPI details (delivery modality, format, other intervention components if relevant); methods of data collection and analysis. Reviewers (MF, EO, KR) extracted data independently, in duplicate. Where necessary, we discussed and consulted the original papers until consensus was reached.

As part of the data extraction phase, and to facilitate the meta-aggregation process, we read and re-read included studies in duplicate (MF, EO, KR) to extract individual findings (i.e., authors’ analytic interpretative statements of qualitative data) and accompanying illustrations (i.e., verbatim participant quotation that exemplifies the finding). Any verbatim analytic statement was eligible to be extracted as a finding, provided an accompanying illustration was available. Where an accompanying illustration was not available, the finding was not included in this review. As per JBI guidelines (24), we (independently and in duplicate) assigned finding and illustration pairings a credibility rating: unequivocal (i.e., illustration supports the finding beyond reasonable doubt and therefore not open to challenge), credible (i.e., illustration lacks clear association with the finding and is therefore open to challenge) or not supported (i.e., illustration does not support the finding).

### 2.5 Risk of bias assessment

Risk of bias assessment was conducted for each eligible study independently by three reviewers (MF, EO, KR) using the JBI Checklist for Qualitative Research (25). In this 10-item tool, each item is rated as: yes, no, unclear, or not applicable. We resolved discrepancies via discussion, re-checking the papers together, and discussion with a fourth author (NP) as required. As per recent guidelines for ensuring review results represent the best available evidence (26) eligible studies were included if they satisfied at least six criteria on the appraisal tool.

### 2.6 Synthesis of results

Qualitative findings were pooled via meta-aggregation (24). Findings, illustrations, and credibility data were exported and printed for repeated reviewing in hard copy and for discussion in duplicate by two authors (EO, KR). Using butchers paper, we manually grouped the printed findings into categories based on our discussions. We first placed findings into categories based on similarity of meaning. Second, we combined similar categories into ‘synthesized findings’, referring to indicatory statements that convey the whole, inclusive meaning of a collection of categories, and which can be used to develop policy and practice recommendations. We then transferred these hard copy synthesized findings back to an Excel spreadsheet for discussion with the wider team. Following team discussion, we prioritized these synthesized findings into conceptual order for presentation in the manuscript.

### 2.7 Assessing confidence in findings

As per JBI guidelines, we used the ConQual approach (27) to establish confidence in each synthesized finding. ConQual argues that confidence in a meta-synthesized finding is determined by the dependability and credibility of the studies and individual findings that comprise it. Confidence ratings range from high, moderate, low, to very low. By default, qualitative studies are initially given a ‘high’ confidence rating, which can be downgraded based on dependability and credibility. Dependability is determined based on performance of each study on items 2-4 and 6-7 of the JBI Checklist for Qualitative Research, with the overall confidence level unchanging if the majority of individual findings are from studies with 4-5 ‘yes’ responses, downgraded one level for majority 2-3 ‘yes’ responses, and downgraded two levels for majority 0-1 ‘yes’ responses. For credibility, where a synthesized finding contains only unequivocal individual findings, no downgrading penalty is applied; however, confidence is downgraded one level if the synthesized finding comprises a mix of unequivocal and credible individual findings.

### 2.8 Reflexivity statement

The overarching qualitative methodology guiding this review was an interpretivist approach, which recognizes subjectivity and reflexivity (28). This approach makes the perspectives and positioning of the authors explicit, ensuring that the impact of researcher lenses on the synthesis and examination of results is transparent. While the components of the SPI should be universal, we acknowledge our positioning in the Australian context, which is associated with a unique set of cultural factors and policy frameworks that influence SPI practices and implementation. It is also important to acknowledge the authors’ backgrounds. Collectively, the research team brings expertise across lived experience, clinical practice, and research. EO is a postdoctoral researcher with expertise in behavioral science and mental health. KR is an experienced mental health nurse and doctoral level health psychologist working in research and education. NP is a professorial level mental health nurse expert and leader in suicide prevention research and education. ML is a Lived Experience academic. AP is a PhD researcher in health and medical sciences and Expert by Experience with the SPI. J-AR is a mental health nurse expert in clinical and senior management. SP is an experienced mental health nurse. MF is a senior suicide prevention researcher.

## 3 Results

### 3.1 Study inclusion

Database searching yielded 1862 results, reduced to 588 after removal of duplicates. Results were screened at the title/abstract level, leaving 60 eligible for full-text screening. One additional article was identified via a correction that appeared in the search results. No further articles were identified through reference list pearling. Twelve eligible studies were critically appraised; two (15, 29) were excluded by the minimum risk of bias threshold, leaving ten studies for inclusion. See

**Figure 1:**
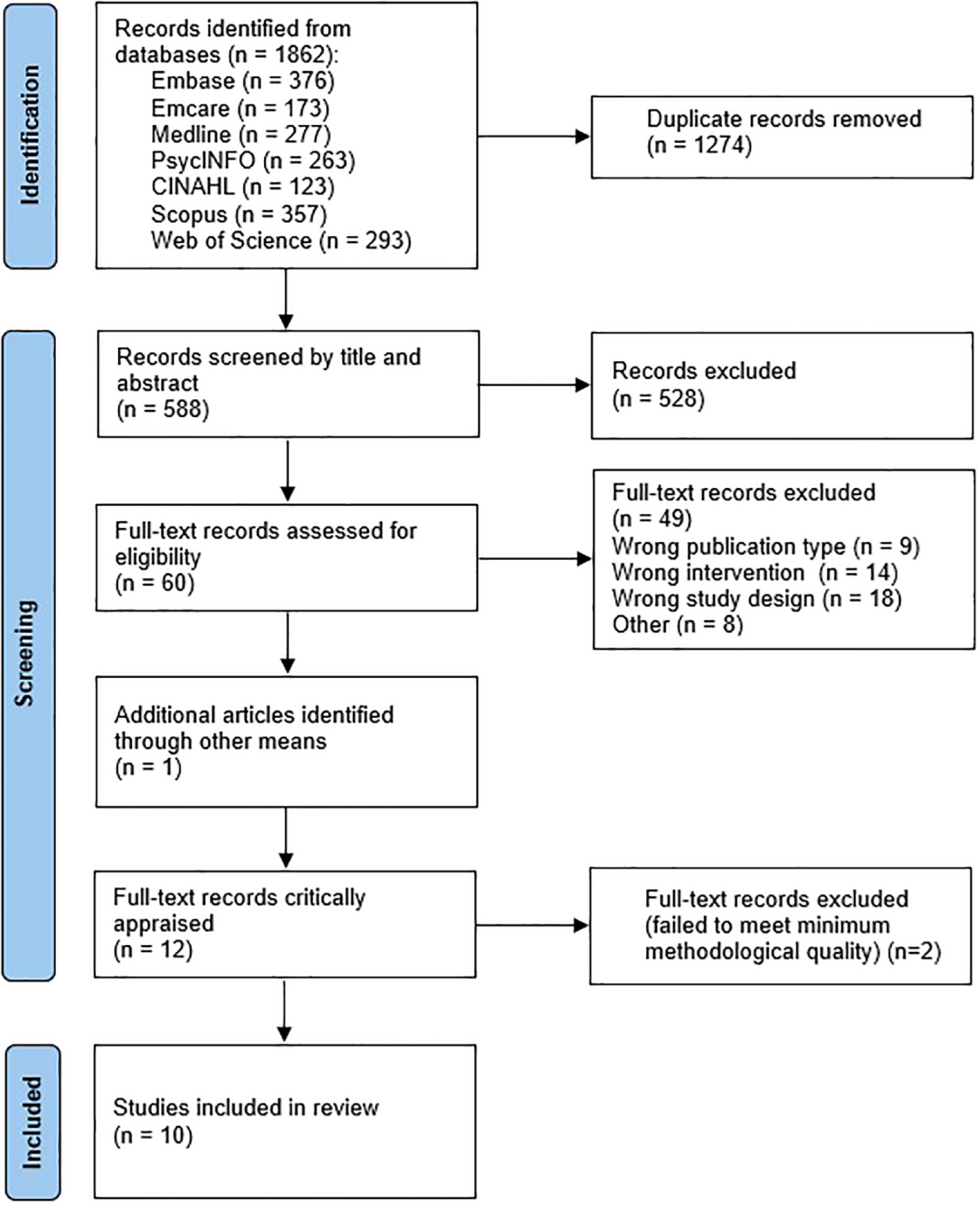
PRISMA flow diagram.

**Figure 1** for the full screening process, and **Supplementary Data Sheet 2** for a list of all ineligible full-text results.

### 3.2 Characteristics of included studies

Included studies were published between 2015 and 2023 and primarily conducted in the United States (n =7). Results for this review are based on data from n = 243 participants (note: this relates to the total number of participants from eligible phases of the included studies). The mean sample size was 24 (range, n=12-50). Across all studies, participants included n = 113 clinicians/staff (n=5 studies), n = 103 adults (including 95 veterans, n=4 studies; and 8 general population, n=1 study), n = 20 adolescents (n=2 studies), and n = 7 support persons (n=2 studies). Eight studies included both female and male participants, two did not report any gender data, and none reported data on other gender identities. Study settings included combined inpatient and outpatient (n=4), outpatient only (n=4), emergency department (n=1), and community services (n=1), with six studies relating to the context of veterans.

Six studies were purely qualitative (10, 30–34), one was mixed methods (35), and while a further three identified as qualitative they also included some minor quantitative aspects (e.g., quantitative measures to collect participant clinical information, 36, 37; or quantification of time spent creating safety plans, 38) but were not considered mixed methods. Most studies (n=8) collected qualitative data via semi-structured individual interviews but focus groups (n=1) and open-ended survey items (n=1) were also used. Studies analyzed qualitative data using thematic analysis (n=4), content analysis (n=2), interpretive phenomenological analysis (n=1), and matrix analysis (n=1). Two studies did not clearly report an analytic method.

There was substantial variability across studies in SPI features, and its role in suicide prevention and mental health care. Studies discussed versions of the SPI including additional components such as text-message and/or telephone follow-up support (31, 35), and the inclusion of support persons (36). Most studies (n=9) used or discussed the SPI as one component of care, alongside other psychological interventions (e.g., individualized, outpatient psychotherapy). The specific format of initial construction, ongoing access, or both, was often unclear. Only three studies described a specific SPI format, including a traditional hard-copy format (33), a mobile phone app-based version (30), and either hard copy or electronic versions (38). There was also a lack of detailed reporting regarding delivery modality, with four studies (30, 31, 33, 38) clearly indicating in-person creation of the SPI, and one describing a group-based SPI delivered online via telehealth (37). Eight studies described who the SPI was co-created with – working with a clinician was the most frequent approach (10, 30, 31, 33, 34, 38), with one study describing construction with a study counselor (35), and another describing a collaborative creation process with other SPI users in a group format (37). See **Table 1** for full characteristics of included studies.

**Table 1:** Characteristics of included qualitative studies (n=10).

Study	Setting (Country)	Phenomena of interest	SPI details	Methods of data collection and analysis^	Participant group/s, sample size and characteristics^
Buus et al. (30)	Outpatient suicide prevention clinic setting (Denmark)	Stakeholder perspectives on a mobile phone app-based version of the SPI	*SPI only:* No, part of short-term, specialized psychotherapy (up to 10 sessions); *Format:* mobile application; *Created with:* clinician; *Modality:* in person.	Focus groups; Thematic analysis	Total n = 26 n = 5 adolescents All female Age range = 14-17yr n = 8 adults 3 female, 5 male Age range = 21-28yr N = 3 relatives 3 female Age range = 48-50yr n = 10 clinicians 9 female, 1 male Age range = 37-60yr
Chesin et al. (31)	Veterans Affairs Medical Center emergency department (USA)	Clinicians’ perceptions of the acceptability and utility of the Safety Planning and Structured Post-Discharge Follow-Up Intervention.	*SPI only:* No, follow-up telephone calls post-discharge (including suicide risk assessment an intervention as needed); *Format*: NR (assume hard copy); *Created with:* clinician; *Modality:* in-person and telephone calls.	Semi-structured individual interviews; Thematic analysis	n = 50 clinicians Gender = NR Age = NR
Czyz et al. (35)*	Outpatient (USA)	Experiences of adolescents who received SPI with automated text-messages	*SPI only:* No, twice-daily text message follow-up for 4 weeks post-discharge; *Format:* NR; *Created with:* Study counselor; *Modality:* NR (assume in person).	Open-ended survey questions; Analysis type not clearly reported.	n = 15 adolescents 11 female, 4 male M(SD) Age = 15.07(1.16) yr
De Beer et al. (36)	Inpatient and outpatient (USA)	Attitudes towards, and experiences of, involving support persons in the SPI process from the perspective of Veterans and their support persons	*SPI only:* No, part of broader VA approach to suicide prevention; *Format:* NR (assume hard copy); *Created with:* NR (assume clinician); *Modality:* NR (assume in person).	Semi-structured individual interviews; Content analysis.	n = 33 n = 29 Veterans 6 female, 23 male M(SD) Age = 47.93 (11.12) yr n = 4 support persons 3 female, 1 male M(SD) Age = 33.25 (3.34) yr
Ferguson et al. (10)	Community services (Australia)	Experiences and perspectives of safety planning from workers who support refugee and asylum seeker clients	*SPI only:* NR *Format:* NR *Created with:* Clinician/staff *Modality:* NR	Semi-structured individual telephone interviews; Reflexive thematic analysis.	n = 12 clinicians/staff 8 female, 4 male M Age = 41 yr (range= 27-64 yr)
Janackovski et al. (32)	Outpatient (Australia)	Clinicians’ views of interventions and therapeutic methods that are effective in reducing suicidality in young people	*SPI only:* No, part of overall response/treatment; *Format:* NR *Created with:* NR *Modality:* NR	Semi-structured individual interviews; Interpretive phenomenological analysis.	n = 12 psychologists 10 female, 2 male M(SD) Age = 30.33 (3.11) yr
Kayman et al. (33)	Inpatient and outpatient (USA)	Veterans’ experiences with the SPI	*SPI only:* No, part of broader VA treatment for suicide; *Format:* hard copy; *Created with:* clinician; *Modality:* in person.	Semi-structured individual interviews; Analysis type not clearly reported.	n = 20 Veterans 9 female, 11 male M Age = 38 yr (range= 23-55 yr)
Levandowski et al. (38)	Inpatient and outpatient (USA)	Clinician perspectives on the value, utility, and impact of the SPI on Veterans’ suicide risk	*SPI only:* No, part of broader VHA approach to suicide prevention; *Format:* hard copy and electronic; *Created with:* clinician; *Modality:* in person.	Semi-structured individual interviews; Thematic analysis.	n = 29 clinicians Gender = NR Age = NR
Matthieu et al. (34)	Inpatient and outpatient (USA)	Veteran feedback on SPI utilization within a Veteran’s health care system	*SPI only:* No, part of broader VHA approach to suicide prevention; *Format:* NR (assume hard copy); *Created with:* Health-care provider or on own; *Modality:* NR (assume in person).	Semi-structured individual interviews; Content analysis.	n= 29 Veterans 6 female, 23 male M(SD) Age = 47.93 (11.12) yr
Patel et al. (37)	Outpatient (USA)	Veteran perspectives on acceptability, feasibility, and impact of a group-based SPI delivered via tele-health	*SPI only:* No, Project Life-Force-telehealth, 10 session safety planning group, using DBT and psychoeducation; *Format:* NR (assume hard copy or electronic); *Created with:* collaboratively during group session, with facilitator; *Modality:* Telephone/video-conferencing group.	Semi-structured individual interviews; Matrix analysis.	n = 17 Veterans 2 female, 15 male M(SD) Age = 50.5 (15.61) yr

^^^ methods and participant details relate only to qualitative components of the included studies (i.e. any quantitative details have not been included here); * Czyz et al. (41) conducted a two-phase study,however only findings from Phase 2 were eligible for extraction in the present review,and therefore details and characteristics of Phase 1 are not recorded here; SPI, Safety Planning Intervention; VA, Department of Veterans Affairs; VHA, Veterans Health Administration; NR, Not reported; M, Mean; SD, Standard deviation; DBT, Dialectical Behavioral Therapy.

### 3.3 Risk of bias within and across studies

Included studies performed well on critical appraisal items related to congruity between research methodology and study methods, as well as ethical research conduct and appropriateness of study conclusions. However, guiding philosophical perspectives were largely unreported, with only one study mentioning this (32), and studies did not consistently meet criteria for reflexivity, with only one study (32) locating the researchers culturally or theoretically, and two studies (10, 32) discussing the influence of the researcher on the research and vice-versa. See **Table 2** for study-level critical appraisal results.

**Table 2:** Critical appraisal of included qualitative studies (n=10).

Study	Q1	Q2	Q3	Q4	Q5	Q6	Q7	Q8	Q9	Q10
Buus et al. (30)	N	Y	Y	Y	Y	N	N	N	Y	Y
Chesin et al. (31)	U	Y	Y	Y	Y	N	N	Y	Y*	Y
Czyz et al. (35)	N	Y	Y	Y	Y	N	N	N	Y	Y
DeBeer et al. (36)	N	Y	Y	Y	Y	N	N	Y	Y	Y
Ferguson et al. (10)	N	Y	Y	Y	Y	N	Y	Y	Y	Y
Janackovski et al. (32)	Y	Y	Y	Y	Y	Y	Y	Y	Y	Y
Kayman et al. (33)	N	Y	Y	Y	Y	N	N	Y	Y	Y
Levandowski et al. (38)	N	Y	Y	Y	Y	N	N	Y	Y	Y
Matthieu et al. (34)	N	Y	Y	Y	Y	N	N	Y	Y*	Y
Patel et al. (37)	N	Y	Y	Y	Y	N	N	Y	Y	Y
Total % Y	10	100	100	100	100	10	20	80	100	100

JBI critical appraisal tool for qualitative research: 1. Is there congruity between the stated philosophical perspective and the research methodology? 2. Is there congruity between the research methodology and the research question or objectives? 3. Is there congruity between the research methodology and the methods used to collect data? 4. Is there congruity between the research methodology and the representation and analysis of data? 5. Is there congruity between the research methodology and the interpretation of results? 6. Is there a statement locating the researcher culturally or theoretically? 7. Is the influence of the researcher on the research,and vice-versa,addressed? 8. Are participants,and their voices,adequately represented? 9. Is the research ethical according to current criteria or,for recent studies,is there evidence of ethical approval by an appropriate body? 10. Do the conclusions drawn in the research report flow from the analysis,or interpretation,of the data?; Y, yes; U, unclear; N, no; * In these studies,the projects were deemed to be performance/quality improvement and “non-research” activities,and therefore no ethical approval was necessary or reported; however,based on the methodological information provided,we deemed the research ethical.

### 3.4 Review findings

Ninety findings (82 unequivocal; 8 credible) related to stakeholders’ experiences of the SPI were extracted and aggregated into 14 unique categories according to similarity of meaning. Four synthesized findings (one moderate confidence and three low confidence) were developed via meta-aggregation. See **Table 3** for a summary of the findings and categories used to create each synthesized finding, and **Supplementary Table 1** for full ConQual results. Complete details of individual findings and illustrations are presented in **Supplementary Table 2**. We provide a narrative description of each synthesized finding and associated categories below.

**Table 3:** Meta-aggregation of findings, categories and synthesized findings.

Finding (credibility rating) (n=90)	Category (n=14)	Synthesized finding (ConQual score) (n=4)
Buy-in for SPI-SFU is possible (U)	Perceived acceptability/usability of the SPI	Synthesized finding 1: Acceptability and positive outcomes associated with the SPI (low) *Stakeholders directly involved in service delivery view the SPI as an acceptable intervention that is associated with numerous benefits. SPI practices are seen to facilitate greater personal awareness regarding the precipitants and expressions of suicide-related distress. Similarly, SPI practices offer opportunities for people experiencing distress to acquire self-regulatory skills and strategies that they can utilize to manage distress more effectively.*
SPI-SFU facilitates veteran connection to follow-up mental healthcare (U)		
SPI-SFU is an acceptable intervention for suicidal veterans (U)		
SPI-SFU mitigates suicide risk among veterans (U)		
Systematic approach to safety planning was considered to be beneficial (U)		
Reported [positive] experience with the plan (U)	SPI practices improve consumers’ understanding of their own suicide-related experiences, and enhance the use of adaptive self-regulatory skills and strategies to manage distress	
Normalizing the client experience (U)		
Perceived benefits of safety planning for the client, particularly its value as a therapeutic tool to address suicidality (U)		
Reminders can help to keep people safe (U)		
Reducing emotional reactivity helped develop reflective capacity and insight (U)		
Safety planning could help the support system be more aware of the young person’s internal difficulties (U)		
To be reminded that they would prefer not to give pain to loved ones (U)		
Veterans’ perceptions on plan construction [awareness] (U)		
Some [clinicians] also thought that safety planning served to prevent unnecessary hospitalizations (U)		
Personalization methods included documenting any suicide method(s) the veteran has considered and what specific steps they will take to stop themselves (U)		
The safety plan supports both providers and veterans (U)		
Identifying warning signs was the most remembered proportion (C)		
Other steps veterans used included identifying warning signs, contacting family or crisis lines, and using coping strategies (U)		
The plan reminded the veteran about personal coping strategies, options for using the plan, and ways to keep their environment safe (C)		
the [SPI] groups helped [consumers] learn to identify warning signs, and understanding the connection between their depression, PTSD and substance use disorder and suicidal thoughts, urges and plans (U)		
[the SPI] helped [consumers] learn how to use distraction to put time between thoughts and actions and identify the need to speak to someone when in a time of crisis (U)		
The collaborative and personalized nature of safety planning (U)	Collaborative, person-centered approach to constructing the safety plan	Synthesized finding 2: Maximizing the effectiveness of the SPI (moderate) *To maximize consumer engagement and effectiveness of the SPI, clinicians should ensure that safety plans are constructed and used in a person-centered way. The collaborative nature of the SPI means that it is an ongoing process over time, wherein therapeutic engagement and support is important. However, organizational factors need to be aligned such that clinicians have a) sufficient time and resources to engage in meaningful, person-centered, therapeutic interactions with consumers, and b) adequate peer and executive support. Digital technology offers opportunities as an alternative delivery modality (i.e., via telehealth) and as a tool to provide adjunct support (e.g., text messages).*
Safety planning: A process tool, not ‘just’ a duty-of-care task (U)		
Veterans’ perceptions on plan construction [collaboration] (U)		
Filling out the safety plan encompassed more than simply filling in the template (U)		
[the SPI] is used for prompting a range of potential strategies if the veteran is having trouble identifying their own ideas (U)		
Strategies to enhance engagement in safety planning. Being flexible and creative (U)	Clinical strategies to improve consumer engagement	
Therapeutic strategies may assist to gently ease into safety planning conversations (U)		
The safety plan should provide the veteran with options and list any scenarios and actions that should be taken while in crisis (U)		
Opportunity to develop personal insight into the typical patterns of their crises (U)	Use of the SPI over time	
A safety plan is an ongoing, living document, revised and revisited as part of ongoing client-worker interactions (U)		
Reported experience with the plan [use over time] (U)		
Safety plans were updated if the patient indicated their stress levels increased, suicidal thoughts had returned or increased, or if they attempted suicide (U)		
Administrative support and integration of service coordination result in successful SPI-SFU implementation (U)	Organizational factors impacting the delivery of the SPI	
The role of interpreters to address language barriers (U)		
Workers must be supported to engage in safety planning (U)		
Challenges with finding sufficient time (U)		
Semi-automatic communication … would not relieve [relatives] of the fundamental uncertainty about their son or daughter’s state of mind, or of their whereabouts (U)	Use of digital technology to deliver and/or support the SPI	
Changing the morning message timing on the weekends might make it more likely that teens will see those messages (U)		
Messages that included humor or memes [negative perceptions] (U)		
Messages that included humor or memes [positive perceptions] (U)		
Messages may be helpful in the postdischarge period by contributing to the improvement in mood and providing a sense of hope (U)		
Majority expressed that they like this function [to request a second daily message] (U)		
Support delivered via an automated text messaging system may be limited (U)		
Text messages could definitely or probably aid in the reduction of crises (U)		
Most [teen consumers] indicated that two messages per day were desired (U)		
The influence of [text] messages may vary based on individual circumstances (U)		
Texts could be helpful by providing coping reminders and supporting adolescents’ transition from hospitalization (U)		
Advantages of joining the group via telehealth (U)		
Group facilitators tried to ensure equitable participation and create space for each group member to speak even though they were not in person (U)		
Reduced burden of making and rescheduling appointments and long waitlists (U)		
The ability to connect with other Veterans having similar experiences and receiving input from others helped [consumers] feel supported (U)		
[the SPI allowed consumers to] open up and disclose their suicidal thoughts to the group or other support persons, share details about emotionally challenging periods during COVID and isolation, and ask for group input on problems that wanted help in solving (U)		
[support persons] indicated they would be willing to be involved in an in-person appointment to meet with the veteran and their provider and to develop the safety plan (U)	Support persons are willing to be involved	Synthesized finding 3: Navigating the involvement of support persons in the SPI (low) *Both consumers and their support persons advocated for the involvement of supportive others in the SPI process. For the consumer, appropriate involvement of support persons in the SPI fosters connection and can circumvent perceptions of burdensomeness. Support persons also act as a valuable resource for recognizing changes in consumers’ states of mind and facilitating earlier help-seeking. This external support function is particularly important when the consumers’ state of mind prohibits independent help-seeking behavior. However, involving support persons in the SPI process should be enacted thoughtfully and in collaboration with the consumer. Support persons can experience secondary distress and may not always support consumers’ preferences for confidentiality and autonomy.*
[support persons] would be devastated if the veteran never shared their thoughts of suicide and then something happened to them (U)		
The safety plan would be helpful (U)		
Additional important aspects of support, including someone they can rely upon (U)	Benefits of support persons’ involvement	
[support persons] indicated they could help by identifying warning signs before the veteran did (U)		
Friends as the most desired source of support (U)		
Involving the [support person] could offer emotional and tangible social support to the veteran (U)		
The support [of friends] as a deterrent to feeling lonely (U)		
Broadening a young person’s support network included involving schools or other supports in the implementation of the safety plan (U)		
Involving family directly in therapy often provided a corrective experience for clients about their perceptions of burdensomeness and increased their sense of belonging (U)		
Sharing it with supportive others, such as friends and family members [as a facilitator] (U)		
Subjective data [from support persons] could improve the provider’s knowledge of circumstances and triggers surrounding the patient’s suicidal ideation or behavior (C)		
Benefits of directly involving family members in safety plans (U)		
Negative consequences of concerned significant other’s involvement (U)	Drawbacks of involving support persons	
[support persons] learning about the veteran’s thoughts of suicide would make them feel worried (U)		
Barriers to implementation and use (U)	The SPI disregarded as unhelpful	Synthesized finding 4: Barriers and limitations associated with the SPI (low) *Although the SPI appears to be widely held in high-regard, clinicians and consumers noted some perceived barriers to engaging with, and benefiting from, SPI practices. Consumers, and even some clinicians, may disregard the SPI as unhelpful and thus resist engaging with it. Some consumers may also be hesitant to engage with safety planning for fear of perceived legal, practical, and emotional implications of disclosure. Moreover, conventional approaches to constructing and using the SPI may present challenges for consumers with limited English language and mental health literacy, or for consumers with differing cultural needs. Even within those who do find general benefit with using the SPI, states of acute distress may temporarily limit consumers’ ability to implement safety planning strategies.*
Depression related lethargy [as a barrier] (C)		
Reported experience with the plan [disregarded as unhelpful] (U)		
Several [clinicians] noted that they did not know if safety planning was effective (U)		
Some veterans felt the safety plan would not be useful (C)		
Barriers to engaging in safety planning - Language and literacy (U)	Barriers to engaging with safety planning	
Barriers to implementation and use … specific to the refugee and asylum seeker context (U)		
Barriers to engaging in safety planning - Organizational Conditions (U)		
The safety plan can be challenging for these clients, particularly given the absence of obvious protective factors (e.g., employment or family), or difficulty accessing mainstream support services (U)		
While some clients are receptive to safety planning conversations, they may be fearful about writing it down (U)		
Lack of privacy as a barrier (U)		
When suicidal thoughts intruded on weekends or at night, and the veteran was unable to reach his/her own doctor, the plan seemed useless, especially if the doctor was perceived as the only reliable source of help (C)		
Privacy concerns before joining PLF-T (U)		
Intense feelings of despair could make it almost impossible to engage with their strategies (U)	Perceived inability to use the SPI during acute distress	
Some strategies could seem too simplistic to users … particularly when they were acutely distressed (U)		
Sometimes [consumers] are overwhelmed so quickly that they felt they had no time to act on their own behalf (U)		
Reluctance to abandon established avoidant coping strategies (U)		
Negative expectations included doubts that the strategies outlined would work (U)		
Mindfulness practices may be harder to engage in times of severe crisis or distress (C)		
Veterans had no desire to use their plan (C)		
Some, however, said it was not helpful to have to discuss and write about warning signs, because this stimulated urges toward self-harm (U)	Limitations of the SPI	
The context in which the safety plan is completed also influenced the shared experience (U)		

U, unequivocal; C, credible; ConQual, confidence rating, with possible ratings: High, Moderate, Low, Very Low.

#### 3.4.1 Synthesized finding 1: Acceptability and positive outcomes associated with the SPI

This synthesized finding comprises 21 individual findings across two categories, revealing that engaging with the SPI is an acceptable intervention, associated with varied benefits to the consumer in the short- and longer-term.

##### 3.4.1.1 Category 1.1 – Perceived acceptability/usability of the SPI

Five findings were located from two studies (31, 38) describing stakeholders’ perspectives on the utility of the SPI. The SPI is deemed an acceptable and even essential intervention by clinicians working with suicidal veterans (31, 38). Clinicians view the SPI as a useful addition to their repertoire, noting that its structured nature can help to facilitate conversations regarding consumers’ emotional states, early warning signs and risk factors (38). Despite initial skepticism about the SPI (31), clinicians describe it as a tool they rely on in everyday practice. For example, one emergency department clinician shared that the SPI assists in engaging individuals with emerging suicidality prior to the onset of suicidal behaviors:

*“[the SPI] has become something we rely on here, which is a testament to how helpful it’s been. [the SPI] allowed us to cast a wider net - catch people before they make an attempt and reach individuals not at high risk. It allowed us to feel like we have more of a handle on a lot more people before they become high risk. We see it as being essential.”* (31 p131).

Further, clinicians who use the SPI with structured telephone follow up stated that it provides a concrete tool to facilitate reduced risk during the transition between inpatient and outpatient settings. For example:

*“I am very satisfied, but partly because it helps facilitate my clinical role as an urgent care psychiatrist in that it provides a bridge between the emergency care and outpatient treatment. I am very pleased with that aspect of that.”* (31 p131).

##### 3.4.1.2 Category 1.2 – SPI practices improve consumers’ understanding of their own suicide-related experiences, and enhance the use of adaptive self-regulatory skills and strategies to manage distress

Sixteen findings from six studies (10, 32–34, 37, 38) of adolescent and adult consumers, and clinicians, form this category describing perceived benefits related to consumers’ ongoing engagement with SPI practices. SPI conversations can broaden consumers’ motivations for keeping themselves safe. This can be achieved by harnessing and amplifying consumers’ awareness of existing reasons for living and generating hope for a more positive future (10), as well as through greater awareness of the emotional pain that would befall consumers’ loved ones in the event of their suicide (33).

SPI processes - supported by reflective, collaborative discussions between consumer and clinician regarding consumers’ lived experiences - helped consumers to develop greater awareness of the character and quality of their emotional states, as well as individual triggers that precipitate the onset and worsening of distress (10, 32, 33, 38). For example, one clinician described how collaborative conversations occurring during the SPI process could help young people to make connections between current distress and earlier triggers:

*“I think having an understanding of why you’re having suicidal thoughts is like the really helpful things a lot of especially young people are like, ‘I don’t know. I just I’m just suicidal. I just feel like shit’. And you really tease that out and like ‘Oh, yeah, you had a fight with your mum. Of course, like that led into this.’ … Some of them do know these things but some of them don’t, and it’s really hard to manage your suicidal thoughts if you don’t realise [sic] what is leading into them. You don’t just have them there’s normally something that happens before that.”* (32 p834).

Another clinician noted that developing greater recognition of their own triggers, warning signs, and effective strategies for emotional regulation allowed consumers to communicate their needs more clearly to supportive others:

*“That the young person clarify in their mind what helps and what doesn’t and what their triggers are and what their warning signs are and then being able to show that to their parents or teachers at school or someone … else that they trust so that they can kind of be prompted to use it”* (32 p835).

Creating a non-judgmental therapeutic environment that normalizes the experience of ambient and acute depressive states may foster consumers’ openness to engage in these difficult and deeply personal conversations (10).

Clinicians described how, over time, consumers learned to independently select and engage ‘lower-level’ self-soothing strategies to avoid deeper states of crisis (38). This perspective was also voiced by consumers in multiple studies:

*“So it helped prepare me a little bit, helped prepare me a little bit more. So in other words you know if, if, if 10 is the highest for the place I most don’t want to be, and 1 is being in this good place, you know uh it kind of helped me to evaluate some things in a way that I can address the issue at 4, 5, or 6 opposed to waiting to get to 8 or 9 to try address it. So I guess having those rules helped me to connect the dots a little better and to come up with um a game plan on how to um deal with things, cope with things a little better.”* (37 p278).

*“I’ve used it to identify when I’m getting into a danger zone and what I can do to help alleviate that”* (34 pe3292).

Taken together, both clinicians and consumers noted that the SPI supported consumers’ autonomy to identify and effectively manage distress.

#### 3.4.2 Synthesized finding 2: Maximizing the effectiveness of the SPI

The second synthesized finding, supported by 32 findings and aggregated into five unique categories, highlights the SPI is perceived to be most effective when it is conducted within a person-centered and collaborative relationship, appropriately involves supportive others, and is integrated in an authentic way within consumers’ ongoing care and personal agency. For both clinicians and consumers, digital technologies may support successful SPI experiences.

##### 3.4.2.1 Category 2.1 – Collaborative, person-centered approach to constructing the safety plan

Five findings from four studies (10, 32, 33, 38) supported this category. Clinicians cautioned that the SPI should not be prescribed by the service provider nor seen as a risk mitigation strategy, but rather constructed collaboratively (10, 32, 38). As one clinician noted:

*“The safety plan is for the client but not for us, so it’s really important that its actually done, you know, really with, pretty much the clients; us facilitating it, but pretty much really the client doing their own safety plan, because it’s for them.”* (10 p3).

Clinicians reported taking approximately 30 minutes to co-construct the initial plan in a collaborative way with meaningful involvement (38). For consumers, the content of the initial plan was arguably less important than the quality of the collaborative therapeutic interaction (33).

##### 3.4.2.2 Category 2.2 – Clinical strategies to improve consumer engagement

This category featured three findings from two studies (10, 34). Staff working with refugees and asylum seekers reported needing to be flexible and creative to ensure that the SPI is accessible and culturally appropriate (10). Clinicians argued that people using the SPI should feel empowered to explore alternative approaches to visualizing and documenting each step, according to the unique consumer needs and preferences (10). Action planning a range of specific steps to take during future crises can help consumers to feel a sense of control in these scenarios, rather than behaving impulsively:

*“Plan out what could possibly happen, and the outcomes and you have it written down then you won’t find yourself doing something spur of the moment.”* (34 pe3292-3293).

##### 3.4.2.3 Category 2.3 – Use of the SPI over time

Four findings from four studies (10, 30, 33, 38) highlight the benefits of ongoing SPI use. Clinicians reported regularly reviewing and updating safety plans, often after consumers had reported recent suicidal ideation or crisis (10, 38). The SPI was seen to provide structure to this process of reflection and, within these discussions, opportunities to adapt the existing plan were explored:

*“I’ll also review them anytime that there is an episode, if someone says, ‘Well, you know. Well, I thought about it.’ [My response would be] ‘So, how’d you cope with that? What did you do?’”* (38 p379).

Consumers described a similar trajectory of adding to or refining their plans following each suicidal crisis (30). This approach was described by one consumer as a process of discovery and personal development:

*“I have a tendency to forget what triggers a crisis. So now, when I experience a crisis and I have no response to it, I write it into the app, where it is not linked to any strategy. And it annoys me that it does not have a strategy, because there needs to be a strategy for everything. So that reminds me to find a strategy, a solution. Over the following weeks you find a strategy so that the next time you are in a crisis thinking “I have felt like this before” then I can go back and “oh, that was what I did. That was how I got through it.”* (30 p56).

This process of addition and refinement may lead to incremental improvements in consumers’ commitment to SPI practices, as well as their capacity to enact safety planning strategies (33).

##### 3.4.2.4 Category 2.4 - Organizational factors impacting delivery of the SPI

Three studies of clinicians (10, 31, 38) provided four findings for this category. Clinicians expressed the need for sufficient time, resources, and support to engage in effective safety planning, with their capacity to create collaborative, person-centered safety plans hampered by insufficient time and competing priorities:

*“If you’re going to expect providers who have a half an hour … to prescribe, look at lab work, follow up on discharge, … medication changes, then [meaningful safety planning] … is challenging.”* (38 p378).

Clinicians acknowledged difficulties establishing staff acceptance of the SPI, suggesting successful implementation of the SPI requires leadership support and clear organizational policies that support best practice (31). Additionally, for consumers with limited English language literacy it is essential for organizations to provide translators or employ clinicians who speak the consumer’s first language (10).

##### 3.4.2.5 Category 2.5 – Use of digital technology to deliver and/or support the SPI

Sixteen individual findings, extracted from three studies (30, 35, 37) described how digital technologies – specifically, text messages and telehealth – could be used to deliver and/or supplement the SPI. Consumers described the impact of automated, personalized text messages as an adjunct to in-person SPI practices (MYPLAN app, 30; 35). For some, the automated text messages were perceived as impersonal and perhaps insufficient depending on the consumers’ individual circumstances (35). However, others found benefit from these support text messages. For example, one consumer shared how this version of the SPI eased their transition out of inpatient care:

*“[you’re] transitioning back into the real world. And it’s good to have a reminder of the skills you learned while you were there. And it’s … like a cushion to help you with your transition back home….when you leave the hospital, a lot of the stuff that you learn there kind of goes out the window. And so, you know, to be reminded about your safety plan and things you might have learned there and things that make you happy is a really good way to kick-start recovery.”* (35 p8).

Finally, consumers of a group-based SPI program delivered via telehealth (Project Life Force-telehealth) voiced that this SPI version bypassed several barriers of traditional in-person mental health care (37). These included practical barriers such as long wait-lists for accessing individual support, as well as social barriers to sharing their lived experiences:

*“I mean, let’s face it, you know, bringing up a conversation about how I feel suicidal is not, is not something you would do at dinner with your friends. So just being in a group with people who were talking about it and sharing those thoughts and those experiences they were having, I mean I just opened up right away. For me it was just good right away, it was just, it was an instant connection to the telehealth group.”* (37 p277).

#### 3.4.3 Synthesized finding 3: Navigating the involvement of support persons in the SPI process

For this synthesis, 15 findings were aggregated into three categories, indicating that including support persons in the SPI process is acceptable and beneficial for the consumer. Some drawbacks might be anticipated relating to confidentiality and support persons experiencing secondary distress.

##### 3.4.3.1 Category 3.1 – Support persons are willing to be involved

Three findings from one study (36) form this category. Support persons of US military veterans described their concern for consumers’ welfare and a desire to support the consumer. Reflecting on their willingness to attend in-person appointments, one support person shared:

*“I would always make time for that. If I had an appointment, I would push my appointment back. My mom, her safety comes first.”* (36 p14).

Being involved in the safety plan also allowed support persons to better understand consumer behavior and support needs (36).

##### 3.4.3.2 Category 3.2 – Benefits of support persons’ involvement

Four studies (32–34, 36) provided ten findings related to the benefits of involving supportive others, such as immediate family members (32–34) friends (36), or a trusted person from extended family, school or broader community (32). From a consumer perspective, involving trusted others was helpful for alleviating feelings of isolation:

*“At least by somebody helping me, that would make me feel as if somebody cared, you know, like if I wasn’t alone in that situation.”* (36 p11).

Clinicians agreed, noting how involving supportive others could provide evidence to contradict consumer feelings of burdensomeness:

*“I think that often shifts when the parents are involved and the family know, and they’re supportive … it’s a lot of that working with everyone, challenging those beliefs and looking at how we can actually demonstrate it they’re not being a burden to getting others involved”* (32 p833).

Consumers and support persons described how sharing the SPI with supportive others offered an important external source of feedback and support (33, 34, 36). Support persons could help recognize warning signs, external triggers, and consumer affect and behavior. As a result, support persons may reduce the help-seeking burden placed on consumers and can provide positive reinforcement when the consumer is doing well (32, 36). Finally, support persons played a vital role in maintaining safer environments, including restricting access to lethal means in the home (34).

##### 3.4.3.3 Category 3.3 - Drawbacks of involving support persons

Potential drawbacks of involving support persons were articulated in two findings from one study (36). Consumers noted that support persons may become overbearing and may share private details with other people without consent. Being involved in the SPI also introduced new emotional challenges for support persons, such as increased worry for the consumer, themselves, and other loved ones who may be affected by suicide-related behaviors:

*“It worries you and you start thinking about what about if it does happen, like you start thinking about your kids and how would you handle it or how, you keep thinking oh my gosh, what do I do, how do I help [the veteran], and you do try—you do try—like I try to make [the veteran] see how blessed [they are] … So that makes me angry, I guess, to think that [the veteran] don’t think about them or me, how would I take it, how would I, you know, how is that going to affect me and the kids and stuff.”* (36 p12).

#### 3.4.4 Synthesized finding 4: Barriers and limitations associated with the SPI

The final synthesized finding was supported by 22 findings, aggregated into four categories, describing a range of challenges associated with the SPI.

##### 3.4.4.1 Category 4.1 - The SPI disregarded as unhelpful

Five findings from four studies (10, 33, 34, 38) described stakeholder skepticism about the utility of the SPI. Clinicians were unsure of the SPI’s effectiveness, both in general and in times of crisis (38). Clinicians also described their experiences with consumers who decline to engage in safety planning at all, perhaps due to stigma attached to suicide-related phenomena (10). Some consumers expressed doubt that any intervention could deter a person with suicidal intent (34). Other consumers doubted the helpfulness of SPI strategies, especially whilst experiencing severe neurovegetative symptoms (33). Finally, one consumer shared the perspective that the SPI was unnecessary:

*“No I didn’t keep it. I didn’t look at it at all. It’s just, the suicide safety plan is one of those things that’s common sense. If you have suicidal thoughts it’s the things you should do – call somebody. It’s not something I should look at it.”* (33 p376).

##### 3.4.4.2 Category 4.2 - Barriers to engaging with safety planning

Barriers to engaging with the SPI were discussed in eight findings across three studies (10, 33, 37). A lack of therapeutic rapport may impair consumer engagement with SPI processes, particularly in situations where consumers lack a regular mental health worker (10). Lack of privacy in consumers’ home environments may interfere with engagement in SPI-based online therapeutic sessions (37), and restrict the use of specific strategies (e.g., singing, 30). Ferguson et al. (10) reported several barriers of relevance to refugee and asylum seeker consumers, particularly related to English language literacy, mental health literacy and/or specific cultural needs. For example:

*“…we assume that all clients will be able to engage with the content that we are discussing and come up with safety plans in their own words but it’s not always the case … there needs to be mental health literacy first before we even ask about suicide.”* (10 p5).

Finally, consumer engagement may be impaired if consumers perceive negative ramifications from disclosing suicidality (e.g., refugee and asylum seeker concerns for visa applications and residency; 10).

##### 3.4.4.3 Category 4.3 - Perceived inability to use the SPI during acute distress

Seven findings from three studies (30, 33, 34) support this category. There was a common perception that, during episodes of severe distress, suicidal ideation dominated conscious awareness and consumers reported feeling unable to consider or initiate behavioral SPI strategies (30, 33, 34):

*“Yes, when you are so far into the red zone. It’s hard to use any tool in that space because your thoughts [about self-harm/suicide] are fixed. There is one thing you want and that is how it is and you forget everything else.”* (30 p57-58).

*“Sometimes I get tunnel vision and I don’t get a chance to make the call but a lot of times I keep it with family so that’s a good thing. Keeping with family is good”* (33 p378)

Given the at-times overwhelming nature of consumers’ distress, some may feel belittled if clinicians suggest ‘simple’ self-care strategies without providing genuine validation of the consumer’s perspective or appropriate justification for strategy suggestions (30).

##### 3.4.4.4 - Category 4.4 Limitations of the SPI

Other limitations of the SPI were noted in two findings from two studies (33, 37). The SPI may be challenging to implement for people with few protective factors (e.g., when consumers cannot identify any support persons or strategies for keeping themselves safe; 37). Finally, the act of formally documenting or reviewing warning signs can itself be a triggering experience for consumers:

*“My trigger is not having my daughter … seeing that makes me want to shoot my foot off”* (33 p376).

## 4 Discussion

Featuring rich data from the perspectives of consumers, clinicians and support persons, this qualitative systematic review provides unique insights regarding the practices and processes perceived to impact on consumers’ experiences with the SPI. Through meta-aggregation, four synthesized findings were produced, with the results indicating that the SPI is a beneficial intervention, enhanced through person-centered collaboration and the involvement of supportive others. However, several perceived limitations impact on perceived acceptability and efficacy, which must be considered by organizations and clinicians involved in service delivery. These findings add an important lived experience lens to SPI literature, complementing previous quantitative studies and reviews of SPI efficacy.

### 4.1 Perceived benefits of the SPI

Consumers, clinicians, and support persons viewed the SPI as broadly acceptable and beneficial for reducing consumers’ suicide risk. These qualitative data concur with previous findings (39), wherein 95% of veterans endorsed the SPI as both acceptable and helpful. In addition, clinicians in the present review perceived SPI practices to be helpful in reducing suicide risk during consumers’ transition from inpatient to home or community settings. This is an important finding, as risk of suicide may be most acute following discharge from psychiatric hospitalization, particularly for those with active suicidal ideation, perceived hopelessness, and history of suicidal behavior (40). Overall, the efficacy of the SPI in helping consumers to reduce suicidal ideation and behavior is supported by both quantitative systematic reviews (17–19) and by the experiences and perspectives synthesized in the present review.

People involved in the SPI also perceived a range of specific benefits that may help to explain the effectiveness of SPI practices. First, person-centered safety planning was seen to facilitate greater consumer autonomy, giving individuals a greater sense of ownership over their own health care. Consumers and clinicians also described how SPI practices helped to increase consumers’ sense of hope by internalizing and valuing their existing reasons for living. The amplification of reasons for living is an important protective mechanism, with reasons for living associated with reduced suicidal ideation and suicide attempts (41). In the present results, reasons for living often included loved ones such as children, partners, family, and friends. As such, greater identification of reasons for living appeared to intersect with an improved sense of connection with supportive others. This fundamental need for connection was maximized when support persons were involved in consumers’ safety planning. Similarly, ongoing engagement with SPI practices supported individuals’ self-efficacy in recognizing early warning signs and engaging self-regulatory coping strategies to interrupt the trajectory of escalating distress. This latter result aligns with recent evidence for growth in suicide-related coping as a key predictor of reduced suicidal ideation during an SPI intervention (16). In sum, the lived experience data synthesized in this review broadly align with some of the psychological mechanisms of effect for the SPI as theorized by Rogers et al. (20). Specifically, these findings add support to Rogers et al.’s (20) suggestions that the SPI promotes autonomy among users, both in initial plan creation and in their choices surrounding whether, when and how to use the plan to keep themselves safe; encourages connection with others (including healthcare services, and friends, family and community), which is a known protective factors against suicide; and builds competence through encouraging individuals to identify personalized support strategies and to practice using these to build confidence over time.

### 4.2 The importance of a collaborative and person-centered approach

Clinicians and consumers strongly recommended a collaborative, person-centered approach to constructing and using the SPI over time. This approach refers to clinicians and consumers working together, sharing decision making and having a balance of power, to develop plans that address the consumer’s unique needs and circumstances (42). Unlike a crisis risk assessment process, which can imply a mechanistic and alienating experience of safety planning, collaborative and person-centered approaches allow a normalizing space for consumers to feel supported and to have voice in exploring suicide-related feelings. Recent quantitative evidence suggests that stronger therapeutic alliance established early in psychotherapy is a key predictor of reductions in suicidal ideation and behavior (43) and this review supports those findings from many consumers using safety plans. Collaborative and person-centered interactions were viewed as essential for helping people in distress to understand and process difficult emotional states, to find meaningful connection with others, and for using their strengths and supports to cope in the future.

Most mental health professionals would recognize the importance of person-centered therapeutic engagement. However, our results highlight a range of organizational barriers impairing clinicians’ ability to use the SPI according to these core principles. Time constraints were the primary barrier impacting clinicians’ perceived ability to conduct person-centered safety planning. Thus, without sufficient organizational support, the SPI may be more likely to be delivered instrumentally with a focus on risk mitigation, rather than in a person-centered and collaborative way.

### 4.3 Influence of consumers’ current state of distress on SPI strategy use

Consumers reported experiences of ‘tunnel vision’ or an inability to consider SPI coping strategies, while enduring acute distress. This finding converges with the understanding that the ability to engage cognitive and/or behavioral self-regulatory coping strategies is diminished during heightened periods of crisis (44). This perceived limitation of SPI utilization further highlights the importance of appropriate and effective methods to work with consumers in deciding to restrict access to lethal means. At an individual level, clinicians and consumers can work collaboratively to make changes to living environments to restrict access to high lethality means should they experience acute and unbearable distress. This part of the planning process should focus on means identified by the consumer that feature in suicidal ideation. Appropriate involvement of support persons may be particularly beneficial in maintaining safe environments and reducing the help seeking burden placed on consumers.

### 4.4 Barriers to SPI engagement

In the present results, the SPI was disregarded as unhelpful by some consumers and clinicians. Similar uncertainty regarding the SPI has recently been documented in a quantitative study, with clinicians doubtful of the effectiveness of safety planning in reducing risk of suicidal behavior (45). As noted by an included study (31), this hesitancy suggests a need for prior education and training about the efficacy, usability, and acceptability of the SPI. Consumers’ fear of disclosure was another barrier to SPI engagement identified in the present results (10). Self-stigma and fear of stigmatized responses to disclosure can deter consumers from seeking help for suicide-related concerns (46), and consumers also report fears of disempowerment from treatment orders under mental health Acts (47). Similar worries may also deter individuals from engaging with interventions such as the SPI.

### 4.5 Recommendations and implications for practice, policy, and future research

The four synthesized findings in this review suggest specific recommendations for practice, policy, and future research. For practice, it is recommended that the SPI is developed via a person-centered and compassionate collaboration, where clinicians are afforded sufficient time (minimum 30 minutes) to develop authentic therapeutic rapport for the person to express their suicidal experiences. Further, to address the transient nature of suicidal thoughts and maximize effectiveness of the safety plan, the SPI should be viewed as a living document that is shared with others (support persons, care providers) and revised regularly. Given that involving support persons appears to enhance the SPI, practitioners should genuinely explore this involvement during the initial safety plan co-construction and at review appointments. Supportive others should receive SPI education with assistance from the clinician and guidance from the consumer regarding how to best provide support.

Regarding policy recommendations, services that use the SPI should include mandatory training for all staff using the SPI, to ensure consistent, evidence-based skill sets and to address the ambivalence of some clinicians identified in this review. Further, there should be clear guidelines and policies for use of the SPI within and across services to ensure continuity of care. For example, the SPI could be proposed as the recommended safety planning instrument in a local context, to be completed before discharge from emergency/inpatient settings and communicated with follow-up care providers as standard practice. Given the diverse contexts in which safety planning is used, there should be flexibility to adapt the SPI to meet diverse consumer needs (e.g., versions in various languages).

Further research is required to address gaps in our understanding of the SPI and how best to support the people who use it. First, the specific processes which assist consumers to reduce suicidal ideation and behavior require further examination. Our findings indicate that SPI practices may enhance consumers’ connection, autonomy, and competence: three of the processes of SPI effect proposed by Rogers et al. (20). Further mixed-methods research is required to investigate causal pathways from specific SPI strategy-use to improved suicide and wellbeing-related outcomes via theorized processes of effect. Greater integration of diverse user experiences is required to inform future SPI adaptations that meet the needs of the specific consumer groups for whom they are designed. In the current review, over half of the included papers related to veterans, their support persons and/or people who work with them. There has been little to no focus on the experiences of safety planning from other priority groups known to experience high rates of suicidality, such as LGBTQIA+ communities (48). Finally, our results reveal a common perception whereby states of acute and severe distress temporarily impair peoples’ capacity to engage in safety planning behaviors. This perceived barrier should be explored in more depth using rigorous qualitative approaches. Research has begun to illuminate the temporal dynamics of suicidal states, often using digital technologies to monitor suicidal distress in real-time (49). Lived experience research will be crucial to develop a greater understanding of how consumers experience the fluctuating and dynamic nature of suicidal states, as well as the relationship between current distress severity and specific SPI strategy use. Such understandings may assist consumers, support persons, clinicians, and researchers to adapt SPI practices to mitigate the onset and worsening of distress, and to improve safety during peak distress.

### 4.6 Strengths and limitations

Our search strategy, study selection procedures and meta-aggregation approach were systematic and thorough. In the JBI approach, findings can only be extracted if accompanied by an illustrative participant quotation. Whilst methodologically rigorous, this may have excluded relevant qualitative data if reported in a different format. There is also substantial scope for improvement in the methodological quality of studies in this area. In the present review, the dependability of included studies was limited due to inconsistent reporting of reflexivity details and guiding methodological frameworks. Three of the four synthesized findings were also downgraded due to a mix of unequivocal and credible findings, resulting in “low” overall confidence ratings. To enhance confidence in future qualitative findings, studies should follow best-practice guidelines for reporting qualitative research. Further, some studies lacked SPI details, such as format and delivery modality. We did not attempt to contact the authors of these papers to seek confirmation of these details. Doing so may have improved the generalizability of findings. However, we do not believe these details to be crucial to the results, as the findings relate more to overall experiences with the SPI, rather than specific features (with the exception that we had one finding category related to digital modalities).

Finally, although one included study indicated a mental health lived experience academic as part of the authorship team (10), none of the included studies explicitly indicate involvement or consultation with people with lived experience of suicidality and/or safety planning in designing or conducting the studies. More high-quality qualitative studies of consumer, support person and clinician perspectives, conceived and conducted collaboratively with people with lived experience of suicidality and safety planning, would advance our understanding of peoples’ experiences of using SPI practices.

## 5 Conclusion

While there is scope for improving the methodological quality of future qualitative SPI research and a need to better understand the causal pathways between SPI use and suicide-related outcomes, the findings from this review indicate that SPI practices are regarded positively from the qualitative perspectives of consumers, support persons and clinicians. This complements what is known about SPI effectiveness from quantitative research, and indicates that the SPI is perceived as acceptable and beneficial, and can be an important strategy to support people experiencing suicide-related distress. Use of the SPI could be strengthened by ensuring that services have sufficient time and resources (including training) for staff to engage in safety planning, as well as pathways for support persons to be involved, and strategies to ensure the SPI is tailored to individual consumer needs. Continuing to prioritize diverse lived experience perspectives of this suicide prevention approach is critical to ensuring that the SPI meets the needs of those using it.

## Data Availability

The original contributions presented in the study are included in the article/**Supplementary Material**. Further inquiries can be directed to the corresponding author.
