# Supplementary material for: The experiences of consumers, clinicians and support persons involved in the safety planning intervention for suicide prevention: a qualitative systematic review and meta-synthesis
Source: Front Psychiatry. 2024 Dec 20;15:1482924. doi: 10.3389/fpsyt.2024.1482924 (PMC11697290; doi:10.3389/fpsyt.2024.1482924)
Supplement: Supplementary file 1 [file DataSheet1.docx]

**Supplementary Data Sheet 1.** Search strategies for all databases

Search strategy run in the Ovid platform (Embase, Emcare, Medline and PsycInfo databases):

1. “safety plan*”.mp

2. exp Suicide/

3. suicid*.mp

4. 2 or 3

5. 1 and 4

6. limit 5 to yr=”2000-current”

7. limit 6 to english language

Search strategy run in CINAHL:

“safety plan*” AND (MH “Suicide+”)

Limiters: published date 20000101-20231231

Narrow by language: English

Search strategy run in Scopus:

( TITLE-ABS-KEY (“safety plan*”) AND TITLE-ABS-KEY (suicide*)) AND PUBYEAR > 1999 AND PUBYEAR <2024 AND ( LIMIT-TO ( LANGUAGE , “English” ) )

Search strategy run in Web of Science:

((TS=(“safety plan*”) AND TS=(suicide*))

Refined by: publication years (2000-2023) and languages (English)
