# Supplementary material for: The experiences of consumers, clinicians and support persons involved in the safety planning intervention for suicide prevention: a qualitative systematic review and meta-synthesis
Source: Front Psychiatry. 2024 Dec 20;15:1482924. doi: 10.3389/fpsyt.2024.1482924 (PMC11697290; doi:10.3389/fpsyt.2024.1482924)
Supplement: Supplementary file 2 [file DataSheet2.docx]

**Supplementary Data Sheet 2.** List of excluded articles

**Studies ineligible following full-text review**

1. Becker-Haimes EM, Schaechter T, Green KL, Bridges J, Jager-Hyman S. Mobile crisis services: A clinician survey of current suicide prevention practices and barriers to care delivery. Community Ment Health J. 2024 Nov 20; 60:562-71.

*Reason for exclusion:* Wrong study design

1. Bettis AH, Donise KR, MacPherson HA, Bagatelas P, Wolff JC. Safety planning intervention for adolescents: Provider attitudes and response to training in the emergency services setting. Psychiatr Serv. 2020 Nov 1;71(11):1136–42.

*Reason for exclusion:* Wrong study design

1. Boudreaux ED, Brown GK, Stanley B, Sadasivam RS, Camargo CA, Miller IW. Computer administered safety planning for individuals at risk for suicide: Development and usability testing. J Med Internet Res. 2017 May 15;19(5):e149.

*Reason for exclusion:* Wrong study design

1. Brenner L, Simpson G. Two promising evidence-based interventions for suicide prevention among veterans with moderate-to-severe TBI. In New Orleans, Louisiana: Brain Injury; 2017. p. 805.

*Reason for exclusion:* Wrong publication type

1. Brenner L. Two promising evidence-based interventions for suicide prevention among veterans with TBI. Arch Phys Med Rehabil. 2016 Oct 1;97(10):e17.

*Reason for exclusion:* Wrong publication type

1. Bruen AJ, Wall A, Haines-Delmont A, Perkins E. Exploring suicidal ideation using an innovative mobile app-Strength Within Me: The usability and acceptability of setting up a trial involving mobile technology and mental health service users. JMIR Ment Health. 2020 Sep 28;7(9):e18407.

*Reason for exclusion:* Wrong intervention

1. Buus N, Juel A, Haskelberg H, Frandsen H, Larsen JLS, River J, et al. User involvement in developing the MYPLAN mobile phone safety plan app for people in suicidal crisis: Case study. JMIR Ment Health. 2019 Apr 16;6(4):e11965.

*Reason for exclusion:* Other - Qualitative data focused on the design of an SPI app. Findings and accompanying illustrations were not available for lived and/or living experiences of direct SPI use.

1. Critchfield KL, Mackaronis JE, Thapa P, Cechak P. A brief overview of safety planning in interpersonal reconstructive therapy. Psychotherapy. 2022 Jun;59(2):168–73.

*Reason for exclusion:* Wrong publication type

1. Darnell D, Pierson A, Whitney JD, Wolkow CA, Dorsey S, Boudreaux ED, Areán PA, Comtois KA. Acute and intensive care nurses’ perspectives on suicide prevention with medically hospitalized patients: Exploring barriers, facilitators, interests, and training opportunities. J Adv Nurs. 2023 Mar 21;79(9):3351-69.

*Reason for exclusion:* Other – Qualitative study of nurses’ perceptions of barriers/facilitators to using SPI and training in the SPI, but prior to using the SPI and/or doing training.

1. Duke S, Macdonald J, Kennedy AJ. Steering Straight: Adapting suicide risk safety planning as a prevention-focused self-management resource for the Australian farming community. Aust J Rural Health. 2023 Feb 10;31(3):471-83.

*Reason for exclusion:* Wrong intervention

1. Durling P, Henni J, Mrozowich D, Rankin J, Barlow A, Grimminck R. A mixed-methods realist analysis of an interdisciplinary simulation intervention for psychiatry residents. Acad Psychiatry. 2022 Feb;46(1):95–105.

*Reason for exclusion:* Wrong intervention

1. Farmer A, Lammas M, Campbell C. A collaborative approach to risk – Primary prevention of self-harm and suicide in first episode psychosis. In: Poster Abstracts. Tokyo, Japan: Early Intervention in Psychiatry; 2014. p. 81.

*Reason for exclusion:* Wrong publication type

1. Finnegan HA, Selwyn CN, Langhinrichsen-Rohling J. ACTively integrating suicide risk assessment into primary care settings. J Ambulatory Care Manage. 2018 Apr;41(2):114–7.

*Reason for exclusion:* Wrong intervention

1. Foo CYS, Gaudiano BA, Friedman-Yakoobian M, Yen S. Acceptance-based parent training intervention for youths with co-occuring psychotic symptoms and suicidal behaviors: A case series from an open trail. 2024 Mar;J Cogn Ther;17:32-52.

*Reason for exclusion:* Wrong intervention

1. Goodman M, Sullivan SR, Spears AP, Dixon L, Sokol Y, Kapil-Pair KN, et al. An open trial of a suicide safety planning group treatment: “Project Life Force”. Arch Suicide Res. 2021 Jul 3;25(3):690–703.

*Reason for exclusion:* Wrong study design

1. Gray NS, Tiller J, Snowden RJ. WARRN – a formulation-based risk assessment procedure for Child and Adolescent Mental Health Services (CAMHS): the view of clinicians. J Forensic Pract. 2019 Nov 11;21(4):228–39.

*Reason for exclusion:* Wrong intervention

1. Higgins A, Doyle L, Downes C, Morrissey J, Costello P, Brennan M, et al. There is more to risk and safety planning than dramatic risks: Mental health nurses’ risk assessment and safety-management practice: Risk Assessment and Safety Planning. Int J Ment Health Nurs. 2016 Apr;25(2):159–70.

*Reason for exclusion:* Wrong intervention

1. Hill RM, Dodd CG, Gomez M, Do C, Kaplow JB. The Safety Planning Assistant: Feasibility and acceptability of a web-based suicide safety planning tool for at-risk adolescents and their parents. Evid-Based Pract Child Adolesc Ment Health. 2020 Apr 2;5(2):164–72.

*Reason for exclusion:* Wrong study design

1. Hughes-Barton D, Skaczkowski G, Fletcher C, Turnbull D, McMahon J, Gunn, KM. What consumers, general practitioners and mental health professionals want: The co-design and prototype testing of a transdiagnostic, acceptance and commitment therapy-based online intervention to reduce distress and promote wellbeing among Australian adults. BMC Public Health. 2023 Sep 14; 1787.

*Reason for exclusion:* Wrong intervention

1. Hutcherson K, Kennard BD, Michaels M, Miles J. Adapting the SAFETY-Acute intervention to improve quality of care for suicidal youth in emergency rooms and medical floors. Evid-Based Pract Child Adolesc Ment Health. 2021 Jul 3;6(3):369–78.

*Reason for exclusion:* Wrong intervention

1. Jeong YW, Chang HJ, Kim JA. Development and feasibility of a safety plan mobile application for adolescent suicide attempt survivors. CIN Comput Inform Nurs. 2020 Aug;38(8):382–92.

*Reason for exclusion:* Wrong study design

1. Kemp K, Pederson CA, Webb M, Williamson S, Elwy AR, Spirito A. Feasibility and acceptability of a brief suicide intervention for youth involved with the family court. Behav Sci Law. 2021 Feb;39(1):26–43.

*Reason for exclusion:* Other - Participants (court staff) were not directly involved with the SPI. Instead, participants gave their thoughts about whether an SPI might be feasible to include in their employment context in the future.

1. Kennard BD, Biernesser C, Wolfe KL, Foxwell AA, Craddock Lee SJ, Rial KV, et al. Developing a brief suicide prevention intervention and mobile phone application: A qualitative report. J Technol Hum Serv. 2015 Oct 2;33(4):345–57.

*Reason for exclusion:* Other - Qualitative data were presented in frequency tables only. No illustrations were available and thus we were not able to extract any data for meta-aggregation.

1. Kimbrel NA, Aho NA, Neal LC, Bernes SA, Beaver TA, Hertzberg JA, Lutrey A, Leto F, Ostiguy W, Cammarata C, Meyer EC, Wilson SM, Dennis MF, Calhoun PS, Beckham JC, Stanley B, Gulliver SB. Development and implementation of web-based safety planning intervention training for firefighter peer support specialists. Crisis. 2024 Mar;45(2):108-17.

*Reason for exclusion:* Wrong study design

1. Labouliere CD, Stanley B, Lake AM, Gould MS. Safety Planning on Crisis Lines: Feasibility, acceptability, and perceived helpfulness of a brief intervention to mitigate future suicide risk. Suicide Life Threat Behav. 2020 Feb;50(1):29–41.

*Reason for exclusion:* Wrong study design

1. Larkin C, Djamasbi S, Boudreaux ED, Varzgani F, Roscoe G, Siddique M, Pietro J, Tulu B. ReachCare mobile apps for patients experiencing suicidality in the emergency department: Development and usability testing using mixed methods. JMIR Form Res. 2023. 7:e41422.

*Reason for exclusion:* Wrong study design

1. Larsen ME, Shand F, Morley K, Batterham PJ, Petrie K, Reda B, et al. A mobile text message intervention to reduce repeat suicidal episodes: Design and development of Reconnecting After a Suicide Attempt (RAFT). JMIR Ment Health. 2017 Dec 13;4(4):e56.

*Reason for exclusion:* Other - Safety planning included as a minor component of a larger intervention design. No qualitative data available specifically for the SPI.

1. Marraccini ME, Pittleman C. Returning to school following hospitalization for suicide-related behaviors: Recognizing student voices for improving practice. Sch Psychol Rev. 2022 May 4;51(3):370–85.

*Reason for exclusion:* Wrong intervention

1. Matthieu M. Correction to: Veteran experiences with suicide ideation, suicide attempt, and social support in safety planning with the Department of Veteran Affairs. Mil Med. 2023 Aug 04. 188(11-12):e3734.

*Reason for exclusion:* Wrong publication type

1. McManama O’Brien KH, Aguinaldo LD, Almeida J, White E. The role of parents in safety planning interventions with suicidal adolescents. Int J Emerg Ment Health Hum Resil. 2016;18(01):727–9.

*Reason for exclusion:* Wrong publication type

1. McManama O’Brien KH, Almeida J, View L, Schofield M, Hall W, Aguinaldo L, et al. A safety and coping planning intervention for suicidal adolescents in acute psychiatric care. Cogn Behav Pract. 2021 Feb;28(1):22–39.

*Reason for exclusion:* Other - Qualitative data were presented in frequency tables only. No illustrations were available and thus we were not able to extract any data for meta-aggregation.

1. Moscardini EH, Hill RM, Dodd CG, Do C, Kaplow JB, Tucker RP. Suicide safety planning: Clinician training, comfort, and safety plan utilization. Int J Environ Res Public Health. 2020 Sep 4;17(18):6444.

*Reason for exclusion:* Wrong study design

1. Nickerson AB, Breux P, Schaffer GE, Samet M. An initial evaluation of the helping students at risk for suicide professional development workshop. Sch Psychol Rev. 2022 May 4;51(3):343–53.

*Reason for exclusion:* Wrong intervention

1. Nuij C, van Ballegooijen W, de Beurs D, de Winter RFP, Gilissen R, O’Connor RC, Smit JH, Kerkhof A, Riper H. The feasibility of using smartphone apps as treatment components for depressed suicidal outpatients. Front Psychiatry. 2022 Sep 27. 13: 971046.

*Reason for exclusion:* Wrong study design

1. O’Grady C, Melia R, Bogue J, O’Sullivan M, Young K, Duggan J. A mobile health approach for improving outcomes in suicide prevention (SafePlan). J Med Internet Res. 2020 Jul 30;22(7):e17481.

*Reason for exclusion:* Other - Qualitative data focused on the design of an SPI app. Findings and accompanying illustrations not available for lived and/or living experiences of direct SPI use.

1. O’Keeffe S, Suzuki M, McCabe R. An ideal-type analysis of people’s perspectives on care plans received from the emergency department following a self-harm or suicidal crisis. Int J Environ Res Public Health. 2023 Oct 04. 20(19):6883.

*Reason for exclusion:* Wrong intervention

1. Pauwels K, Aerts S, Muijzers E, De Jaegere E, van Heeringen K, Portzky G. BackUp: Development and evaluation of a smart-phone application for coping with suicidal crises. Jiménez-Murcia S, editor. PLOS ONE. 2017 Jun 21;12(6):e0178144.

*Reason for exclusion:* Wrong study design

1. Skovgaard Larsen JL, Frandsen H, Erlangsen A. MYPLAN - a mobile phone application for supporting people at risk of suicide. Crisis. 2016 May;37(3):236–40.

*Reason for exclusion:* Wrong publication type

1. Snowden RJ, Holt J, Simkiss N, Smith A, Webb D, Gray NS. WARRN – a formulation-based risk assessment process: its implementation and impact across a whole country. J Ment Health Train Educ Pract. 2019 Oct 24;14(6):399–410.

*Reason for exclusion:* Wrong intervention

1. Spangler DA, Muñoz RF, Chu J, Leykin Y. Perceived utility of the internet-based safety plan in a sample of internet users screening positive for suicidality. Crisis. 2020 Mar;41(2):146–9.

*Reason for exclusion:* Wrong study design

1. Spangler, DA, Muñoz RF, Chu J, Leykin Y. Utilization of an automated internet-based safety plan. J Technol Behav Sci. 2020 June. 5(2):129-38.

*Reason for exclusion:* Wrong study design

1. Stanley B, Chaudhury SR, Chesin M, Pontoski K, Bush AM, Knox KL, et al. An emergency department intervention and follow-up to reduce suicide risk in the VA: Acceptability and effectiveness. Psychiatr Serv. 2016 Jun;67(6):680–3.

*Reason for exclusion:* Other - Qualitative data were presented in frequency tables only. No illustrations were available and thus we were not able to extract any data for meta-aggregation.

1. Thompson E, Nail M, Velotta S, Zaidi Z, Chandler M, Guthrie K, Yen S. Navigating safety in the context of psychosis: Using qualitative themes and stakeholder feedback to adapt safety planning for high-risk adolescents. Early Interv Psychia. 2023. 17(Supp 1):71.

*Reason for exclusion:* Wrong publication type

1. Thompson EC, Nail M, Yen S. Suicide risk and psychotic experiences: Considerations for safety planning with adolescents. R I Med J. 2013. 105(4):26-30.

*Reason for exclusion:* Wrong study design

1. Vijayakumar L, Mohanraj R, Kumar S, Jeyaseelan V, Sriram S, Shanmugam M. CASP – An intervention by community volunteers to reduce suicidal behaviour among refugees. Int J Soc Psychiatry. 2017 Nov 1;63(7):589–97.

*Reason for exclusion:* Wrong intervention

1. Whitmyre ED, Esposito-Smythers C, Goldberg DG, Scalzo G, Defayette AB, Lopez R. Implementation of an electronic safety plan within a measurement feedback system. Evid-Based Pract Child Adolesc Ment Health. 2023. 8(4):541-548.

*Reason for exclusion:* Wrong study design

1. Whitmyre ED, Esposito-Smythers C, Goldberg DG, Scalzo G, Defayette AB, Lopez R. Impact of a virtual suicide safety planning training on clinician knowledge, self-efficacy, and use of safety plans in community mental health clinics. Arch Suicide Res. 2024. 28(1):428-37.

*Reason for exclusion:* Wrong study design

1. Wilson MP, Waliski A, Thompson Jr RG. Feasibility of peer-delivered suicide safety planning in the emergency department: Results from a pilot trial. Psychiatr Serv. 2022 Oct. 73(10):1087-93.

*Reason for exclusion:* Wrong study design

1. Zuckerbrot RA, Cheung AH, Jensen PS, Stein REK, Laraque D, and the GLAD-PC Steering Group. Guidelines for Adolescent Depression in Primary Care (GLAD-PC): I. Identification, Assessment, and Initial Management. Pediatrics. 2007 Nov 1;120(5):e1299–312.

*Reason for exclusion:* Wrong publication type

**Studies excluded after critical appraisal**

1. Chesin MS, Sonmez CC, Benjamin-Phillips CA, Beeler B, Brodsky BS, Stanley B. Preliminary effectiveness of adjunct mindfulness-based cognitive therapy to prevent suicidal behavior in outpatients who are at elevated suicide risk. Mindfulness. 2015 Dec;6(6):1345–55.

*Reason for exclusion:* Satisfied fewer than six of ten critical appraisal criteria (score achieved = 4/10)

1. Melvin GA, Gresham D, Beaton S, Coles J, Tonge BJ, Gordon MS, et al. Evaluating the feasibility and effectiveness of an australian safety planning smartphone application: A pilot study Within a Tertiary Mental Health Service. Suicide Life Threat Behav. 2019;49(3):846–58.

*Reason for exclusion:* Satisfied fewer than six of ten critical appraisal criteria (score achieved = 4/10)
