# Supplementary material for: The experiences of consumers, clinicians and support persons involved in the safety planning intervention for suicide prevention: a qualitative systematic review and meta-synthesis
Source: Front Psychiatry. 2024 Dec 20;15:1482924. doi: 10.3389/fpsyt.2024.1482924 (PMC11697290; doi:10.3389/fpsyt.2024.1482924)
Supplement: Supplementary file 3 [file Table1.docx]

**Supplementary Table 1.** ConQual summary of findings

| **Synthesized finding** | **Type of research** | **Dependability** | **Credibility** | **ConQual score** | **Comments** |
| --- | --- | --- | --- | --- | --- |
| Synthesized finding 1: Acceptability and positive outcomes associated with the SPI | Qualitative | Moderate (downgraded 1 level) | Low  (downgraded 1 level) | Low | Dependability: 16 findings scored 3/5, three scored 4/5, two scored 5/5, so rating downgraded one level to moderate.  Credibility: 19 findings ranked unequivocal, 2 ranked credible, so rating downgraded one level to Low. |
| Synthesized finding 2: Maximizing the effectiveness of the SPI | Qualitative | Moderate (downgraded 1 level) | Moderate  (no change) | Moderate | Dependability: 25 findings scored 3/5, six scored 4/5, one scored 5/5, so rating downgraded one level to moderate.  Credibility: All 32 findings ranked credible, so rating remained unchanged at Moderate. |
| Synthesized finding 3: Navigating the involvement of support persons in the SPI process | Qualitative | Moderate (downgraded 1 level) | Low  (downgraded 1 level) | Low | Dependability: 13 findings scored 3/5, two scored 5/5, so rating downgraded one level to moderate.  Credibility: 14 findings ranked unequivocal, 1 credible, so rating downgraded one level to Low. |
| Synthesized finding 4: Barriers and limitations associated with the SPI | Qualitative | Moderate (downgraded 1 level) | Low  (downgraded 1 level) | Low | Dependability: 16 findings scored 3/5, six scored 4/5, so rating downgraded one level to moderate.  Credibility: 17 findings ranked unequivocal, 5 credible, so rating downgraded one level to Low. |

Range of possible ConQual scores = High, Moderate, Low, Very Low.
