# Supplementary material for: The experiences of consumers, clinicians and support persons involved in the safety planning intervention for suicide prevention: a qualitative systematic review and meta-synthesis
Source: Front Psychiatry. 2024 Dec 20;15:1482924. doi: 10.3389/fpsyt.2024.1482924 (PMC11697290; doi:10.3389/fpsyt.2024.1482924)
Supplement: Supplementary file 4 [file Table2.docx]

**Supplementary Table 2.** Individual finding and illustration pairings (n=90) from each included study (n=10)

| **Buus N, Erlangsen A, River J, Andreasson K, Frandsen H, Larsen JLS, et al. Stakeholder perspectives on using and developing the myplan suicide prevention mobile phone application: A focus group study. Arch Suicide Res. 2020;24(1):48–63.** | |
| --- | --- |
| Finding 1 | Opportunity to develop personal insight into the typical patterns of their crises (U) |
| Illustration | "I have a tendency to forget what triggers a crisis. So now, when I experience a crisis and I have no response to it, I write it into the app, where it is not linked to any strategy. And it annoys me that it does not have a strategy, because there needs to be a strategy for everything. So that reminds me to find a strategy, a solution. Over the following weeks you find a strategy so that the next time you are in a crisis thinking “I have felt like this before” then I can go back and “oh, that was what I did. That was how I got through it.” (p.56) |
| Finding 2 | Some strategies could seem too simplistic to users...particularly when they were acutely distressed (U) |
| Illustration | "Some people feel quite belittled by our "hold on" strategies that seem banal if you haven't trained them. They believe they have a completely overwhelming problem and we suggest taking a bath or going for a walk or something like that. What is the rationale for doing it? If you don't understand the rationale you may feel belittled by relatively simple solutions that can give relief. And it can create trouble if you introduce them [such strategies] while they are in that state." (p.57) |
| Finding 3 | Intense feelings of despair could make it almost impossible to engage with their strategies (U) |
| Illustration | "Yes, when you are so far into the red zone. It’s hard to use any tool in that space because your thoughts [about self-harm/suicide] are fixed. There is one thing you want and that is how it is and you forget everything else." (pp.57-58) |
| Finding 4 | Semi-automatic communication... would not relieve [relatives] of the fundamental uncertainty about their son or daughter’s state of mind, or of their whereabouts (U) |
| Illustration | "You could write the prewritten messages yourself. Our kids could write four messages and choose which one matched their current state of mind. But you will always be in a state of alarm if you know that you have a child feeling bad somewhere, no matter whether you have an app or you don’t." (p. 58) |
| **Chesin MS, Stanley B, Haigh EAP, Chaudhury SR, Pontoski K, Knox KL, et al. Staff views of an emergency department intervention using safety planning and structured follow-up with suicidal veterans. Arch Suicide Res. 2017;21(1):127–37.** | |
| Finding 5 | SPI-SFU is an acceptable intervention for suicidal veterans (U) |
| Illustration | ‘‘I am very satisfied, but partly because it helps facilitate my clinical role as an urgent care psychiatrist in that it provides a bridge between the emergency care and outpatient treatment. I am very pleased with that aspect of that.’’ (p.131) |
| Finding 6 | SPI-SFU mitigates suicide risk among veterans (U) |
| Illustration | ‘‘[SPI-SFU] has become something we rely on here, which is a testament to how helpful it’s been. [SPI-SFU] allowed us to cast a wider net—catch people before they make an attempt and reach individuals not at high risk. It allowed us to feel like we have more of a handle on a lot more people before they become high risk. We see it as being essential.’’ (p.131) |
| Finding 7 | SPI-SFU facilitates veteran connection to follow-up mental healthcare (U) |
| Illustration | ‘‘I have been very pleased with [SPI-SFU]. I’ve really enjoyed being a part of it. I feel like it’s really been a very valuable service that we’re been able to provide for veterans. It provides many of us, including myself, with a certain measure of security for patients that we either discharge from ED or patients that we see in the walk-in clinic. Even the walk-in patients may not get an appointment in the mental health clinic for 2-4 weeks, and not infrequently, they miss that appointment. Having that follow-up component makes a difference and helps people get engaged. It makes me feel better about making those referrals or discharging people from the ED, because I know that mechanism is in place and that there will be some follow-up or a handoff accomplished.’’ (p.131) |
| Finding 8 | Buy-in for SPI-SFU is possible (U) |
| Illustration | ‘‘I’m going to be honest with you. When it first started, I was like, ‘Are you serious? Do you really think a safety plan is going to make that much of a difference?’ But now, I do believe that it does make a difference. It does make the Veteran feel better about things, and it does make them feel like somebody truly cares about them . . . With this in place, it is showing them that no, somebody does care, and somebody is going to follow you. And, they’re going to make sure that you are okay, and if you’re not, we’re going to do some type of intervention.’’ (p.132) |
| Finding 9 | Administrative support and integration of service coordination result in successful SPI-SFU implementation (U) |
| Illustration | ‘‘It’s not an overnight process to get people to buy-in. I would say it helps to have top-down support, the kind of administrative support. Mandates are tricky in some ways, but in some ways they force buy-in. People start to buy-in once they’re forced to.’’ (p.132) |
| **Czyz EK, Arango A, Healy N, King CA, Walton M. Augmenting safety planning with text messaging support for adolescents at elevated suicide risk: Development and acceptability study. J Med Internet Res Ment Health. 2020;7(5):17345.^** | |
| Finding 10 | Texts could be helpful by providing coping reminders and supporting adolescents’ transition from hospitalization (U) |
| Illustration | "[you’re] transitioning back into the real world. And it’s good to have a reminder of the skills you learned while you were there. And it’s...like a cushion to help you with your transition back home. ...when you leave the hospital, a lot of the stuff that you learn there kind of goes out the window. And so, you know, to be reminded about your safety plan and things you might have learned there and things that make you happy is a really good way to kick-start recovery." |
| Finding 11 | Messages may be helpful in the postdischarge period by contributing to the improvement in mood and providing a sense of hope (U) |
| Illustration | "Getting those texts kind of made me feel better...on a day that was going rough, and I would look at it and do what it said...it just kind of made me feel better. Knowing that I can do this, and that people are there wanting to help me and get me better." |
| Finding 12 | Support delivered via an automated text messaging system may be limited (U) |
| Illustration | "[some people] could definitely benefit a lot from it, just some people don’t like automated text messages. So, like, texts from real people." |
| Finding 13 | Text messages could definitely or probably aid in the reduction of crises (U) |
| Illustration | "Definitely...just getting those tips and coping strategies, like little nudges, to remind myself of things I need to do to stay healthy and happy." |
| Finding 14 | The influence of [text] messages may vary based on individual circumstances (U) |
| Illustration | "even though it's helpful, I'm not sure if for some people it would be enough" |
| Finding 15 | Most [teen consumers] indicated that two messages per day were desired (U) |
| Illustration | "two per day was the sweet spot." |
| Finding 16 | Changing the morning message timing on the weekends might make it more likely that teens will see those messages (U) |
| Illustration | "I was never up that early [on weekends]...I would have other notifications on my phone and I just wouldn’t notice it because I just wasn’t up." |
| Finding 17 | Messages that included humor or memes [positive perceptions] (U) |
| Illustration | "It was nice to just get a laugh...Cause sometimes if I was having a bad time and something like that would pop up, you know it was kind of funny and I was just happy for a second. And just, like, realize that it’s not all bad." |
| Finding 18 | Messages that included humor or memes [negative perceptions] (U) |
| Illustration | "The one that looks like a meme...I found it more annoying than anything." |
| Finding 19 | Majority expressed that they like this function [to request a second daily message] (U) |
| Illustration | "I liked that it was kind of a surprise message and that it was optional" |
| **DeBeer BB, Matthieu MM, Kittel JA, Degutis LC, Clafferty S, Qualls N, et al. Quality improvement evaluation of the feasibility and acceptability of adding a concerned significant other to safety planning for suicide prevention with veterans. J Ment Health Couns. 2019;41(1):4–20.** | |
| Finding 20 | Friends as the most desired source of support (U) |
| Illustration | "I’ve got a friend and he knows what’s going on and he understands." (p.9) |
| Finding 21 | Additional important aspects of support, including someone they can rely upon (U) |
| Illustration | "They’re there for me whenever I need them. ... It’s good to have some people you can trust, and I only have so many people." (p.10) |
| Finding 22 | The support [of friends] as a deterrent to feeling lonely (U) |
| Illustration | "At least by somebody helping me, that would make me feel as if somebody cared, you know, like if I wasn’t alone in that situation." (p.11) |
| Finding 23 | Involving the [support person] could offer emotional and tangible social support to the veteran (U) |
| Illustration | "So that way when those warning signs pop up, they’ll know that it’s time to intervene." (p.11) |
| Finding 24 | Negative consequences of concerned significant other’s involvement (U) |
| Illustration | "The cons would just be that once one person would know, the entire family would know." (p.12) |
| Finding 25 | [support persons] learning about the veteran’s thoughts of suicide would make them feel worried (U) |
| Illustration | "It worries you and you start thinking about what about if it does happen, like you start thinking about your kids and how would you handle it or how, you keep thinking oh my gosh, what do I do, how do I help [the veteran], and you do try—you do try—like I try to make [the veteran] see how blessed [they are] … So that makes me angry, I guess, to think that [the veteran] don’t think about them or me, how would I take it, how would I, you know, how is that going to affect me and the kids and stuff." (p.12) |
| Finding 26 | [support persons] would be devastated if the veteran never shared their thoughts of suicide and then something happened to them (U) |
| Illustration | "I think that would be devastating to me. … I would rather know that was going on so that I could feel like I did something." (p.13) |
| Finding 27 | The safety plan would be helpful (U) |
| Illustration | "When [the veteran] first got out of the military, it felt as if it wasn’t anything there that we can use. We didn’t know about anything, so having the safety plan, especially when they’re getting out …because if you’re in the dark and you start seeing these things, you might be like, why are you acting like this? … So having information would have been the best thing for us." (p.13) |
| Finding 28 | [support persons] indicated they could help by identifying warning signs before the veteran did (U) |
| Illustration | "me looking to notice the signs because sometimes [the veteran] may not realize it, but looking in at it I can see it. … Yeah, just watching [them], oh, something isn’t right. … You want to at first, of course, ask questions and then see where we need to go with it." (p.13) |
| Finding 29 | [support persons] indicated they would be willing to be involved in an in-person appointment to meet with the veteran and their provider and to develop the safety plan (U) |
| Illustration | "I would always make time for that. If I had an appointment, I would push my appointment back. My mom, her safety comes first." (p.14) |
| **Ferguson M, Posselt M, McIntyre H, Loughhead M, Kenny MA, Mau V, et al. Staff perspectives of safety planning as a suicide prevention intervention for people of refugee and asylum-seeker background: A qualitative investigation. Crisis- J Crisis Interv Suicide Prev. 2022;43(4):331–8.** | |
| Finding 30 | The collaborative and personalized nature of safety planning (U) |
| Illustration | "The safety plan is for the client but not for us, so it’s really important that its actually done, you know, really with, pretty much the clients; us facilitating it, but pretty much really the client doing their own safety plan, because it’s for them." (p.3) |
| Finding 31 | A safety plan is an ongoing, living document, revised and revisited as part of ongoing client-worker interactions (U) |
| Illustration | "...reminding them every time as well about their safety plan, so that. . .there is more of the chance of them to remember it when they are in distress." (p.4) |
| Finding 32 | Perceived benefits of safety planning for the client, particularly its value as a therapeutic tool to address suicidality (U) |
| Illustration | ". . .like really clearly identifying that going to the beach is something that makes [the client] happy. He might have already known that, but it might not have sort of been identified that he did know that." (p.4) |
| Finding 33 | Normalizing the Client Experience (U) |
| Illustration | "...it’s helping the person I am working with to be more assertive, understanding of why and how, you know, the triggers that make him to feel more low, then he’s aware of those moments, so we are normalizing not just the moment where he’s feeling very low but also the whole process. . .I think that he feels more safe to have the discussion where before. . .he was feeling very embarrassed." (p.4) |
| Finding 34 | Reminders Can Help to Keep People Safe (U) |
| Illustration | ". . .the result can be very big and with some clients it helps them when they get very emotional, they want to just see something that changes their mind and think about a different picture." (p.4) |
| Finding 35 | Barriers to implementation and use (U) |
| Illustration | "When I first introduced the word “safety plan” to the client, because there was a suicide idea, they said “no”. . .they didn’t want to do it" (p.4) |
| Finding 36 | Barriers to implementation and use...specific to the refugee and asylum seeker context (U) |
| Illustration | "...[clients] know that we would convey some information about them back to immigration and they don’t know what information. . .because it is a little bit, sort of vague, as to how much immigration can take of our information. And so they would sometimes worry. . . “how is this going to affect my visa?” . . . "maybe I will not tell her that [I’m suicidal] because maybe then they’ll think I’m a risk to society and then they won’t want me here. . .?”" (p.4) |
| Finding 37 | Barriers to Engaging in Safety Planning - Language and Literacy (U) |
| Illustration | "...we assume that all clients will be able to engage with the content that we are discussing and come up with safety plans in their own words but it’s not always the case. . . there needs to be mental health literacy first before we even ask about suicide." (p.5) |
| Finding 38 | Barriers to Engaging in Safety Planning - Organisational Conditions (U) |
| Illustration | "...a client might not necessarily have a one-on-one case worker. . .they may be seen by any available case worker. . .so it’s difficult to maintain a relationship or to build up a rapport to have such a difficult and really vulnerable conversation. " (p.5) |
| Finding 39 | The safety plan can be challenging for these clients, particularly given the absence of obvious protective factors (e.g., employment or family), or difficulty accessing mainstream support services (U) |
| Illustration | "...unfortunately, what we find is a lot of the services that [you] might be able to rely on normally, like the, you know, the call-back services and things like that, if our clients don’t have a fair level of English, sometimes those services can be difficult for them to navigate." (p.5) |
| Finding 40 | While some clients are receptive to safety planning conversations, they may be fearful about writing it down (U) |
| Illustration | "Some others don’t prefer to write, some they just prefer to hold those thoughts in their minds, maybe they don’t want to write them down because somebody will see them. . .some other clients will say ’no way, we can’t write it down because that makes it more real’." (p.5) |
| Finding 41 | Strategies to Enhance Engagement in Safety Planning. Being flexible and creative (U) |
| Illustration | "...to make those safety plans culturally, linguistically and I think literacy, considering the literacy levels of the client, making them appropriate for the client. " (p.5) |
| Finding 42 | Therapeutic strategies may assist to gently ease in to safety planning conversations (U) |
| Illustration | "...after he said “no,” I then said to him, “would you be able to tell me what reasons you have to live?” And he answered it. . .And then I started to chat about the supports he had." (p.5) |
| Finding 43 | The role of interpreters to address language barriers (U) |
| Illustration | "...an interpreter needs to be available to ensure that communication is clear and meaningful." (p.5) |
| Finding 44 | Workers must be supported to engage in safety planning (U) |
| Illustration | "showing alternative ways to do safety plans" (p.5) |
| **Janackovski A, Deane FP, Hains A. Psychotherapy and youth suicide prevention: An interpretative phenomenological analysis of specialist clinicians’ experiences. Clin Psychol Psychother. 2021;28(4):828–43.** | |
| Finding 45 | Safety planning: A process tool, not ‘just’ a duty-of-care task (U) |
| Illustration | "Um, unhelpful? … Coming from a place of being totally risk adverse, and just box-ticking, I think is really unhelpful, and not hearing out the young person and being mostly concerned with just getting a safety plan." (p.832) |
| Finding 46 | Involving family directly in therapy often provided a corrective experience for clients about their perceptions of burdensomeness and increased their sense of belonging (U) |
| Illustration | "I think that often shifts when the parents are involved and the family know, and they're supportive … it's a lot of that working with everyone, challenging those beliefs and looking at how we can actually demonstrate it they're not being a burden to getting others involved" (p.833) |
| Finding 47 | Broadening a young person's support network included involving schools or other supports in the implementation of the safety plan (U) |
| Illustration | "… they felt that they couldn't talk to their—their parents about it, because they [parents] had so many things that were going on. So, in that particular situation, I looked at the, kind of, broader community, the broader family, is there somebody else? Is there an aunty, is there any uncle, is there somebody at school, is there a friend's parent, is there somebody else that you can get that support from, … and they found, um, an aunty that was really supportive, and that's who we, kind of, involved in safety planning, and that connectedness, kind of, really helped them, and then the aunty was able to talk to the parents about what was happening, and - and, kind of, share that load a little bit as well … Young people don't live in a bubble. We can't treat them in isolation, we have to treat them with the other systems that are in place around them, … I guess, the tricky part of that is that young people inherently are very private, they don't like including other people, um, they like being able to ‘sort their own shit out on their own’, as one young person has said to me … so it's trying to support them to feel comfortable in including those other people, um, it—it just—in my experience, it doesn't work when you just try to do it with the young person alone." (p.833-834) |
| Finding 48 | Reducing emotional reactivity helped develop reflective capacity and insight (U) |
| Illustration | "I think having an understanding of why you're having suicidal thoughts is like the really helpful things a lot of especially young people are like, ‘I don't know. I just I'm just suicidal. I just feel like shit’. And you really tease that out and like ‘Oh, yeah, you had a fight with your mum. Of course, like that led into this.’ … Some of them do know these things but some of them don't, and it's really hard to manage your suicidal thoughts if you don't realise what is leading into them. You don't just have them there's normally something that happens before that." (p.834) |
| Finding 49 | Safety planning could help the support system be more aware of the young person's internal difficulties (U) |
| Illustration | "That the young person clarify in their mind what helps and what doesn't and what their triggers are and what their warning signs are and then being able to show that to their parents or teachers at school or someone … else that they trust so that they can kind of be prompted to use it" (p.835) |
| **Kayman DJ, Goldstein MF, Dixon L, Goodman M. Perspectives of suicidal veterans on safety planning: Findings from a pilot study. Crisis- J Crisis Interv Suicide Prev. 2015;36(5):371–83.** | |
| Finding 50 | Veterans' Perceptions on Plan Construction [awareness] (U) |
| Illustration | "An aspect of the initial plan construction encounter that was cited as helpful was “just to think about who I’ll be hurting and who I can contact to help me through a situation.”" (p.376) |
| Finding 51 | Veterans' Perceptions on Plan Construction [collaboration] (U) |
| Illustration | "focusing more on the collaborative process than on content, a veteran said the most helpful aspect of the encounter was talking with the doctor and seeing that the doctor was “concerned enough to make one” (make a safety plan)." (p. 476) |
| Finding 52 | Some, however, said it was not helpful to have to discuss and write about warning signs, because this stimulated urges toward self-harm (U) |
| Illustration | "My trigger is not having my daughter… seeing that makes me want to shoot my foot off" (p.376) |
| Finding 53 | To be reminded that they would prefer not to give pain to loved ones (U) |
| Illustration | "When you do something like that you harm others too" (p.376) |
| Finding 54 | Negative expectations included doubts that the strategies outlined would work (U) |
| Illustration | "I said I get a little bit of comfort if I’m suicidal maybe I’ll go play guitar or keyboard but that’s BS. That’s really BS because when I’m really at that level of depression when I’m bedridden considering killing myself I’m not going to stand up and grab the guitar. I’m just going to lay there." (p.376) |
| Finding 55 | Reported experience with the plan [use over time] (U) |
| Illustration | "It doesn’t work if you don’t use it. And I’m learning I have to use it." (p.376) |
| Finding 56 | Reported [positive] experience with the plan (U) |
| Illustration | "I don’t go over every detail of it but I’m focused on the parts that are most applicable during that emotion." (p.376) |
| Finding 57 | Reported experience with the plan [disregarded as unhelpful] (U) |
| Illustration | "No I didn’t keep it. I didn’t look at it at all. It’s just, the suicide safety plan is one of those things that’s common sense. If you have suicidal thoughts it’s the things you should do – call somebody. It’s not something I should look at it." (p.376) |
| Finding 58 | Lack of privacy as a barrier (U) |
| Illustration | "Well I am having a little bit of difficulty, it’s like a snag with the plan, because I’m enforcing what I need to do but it’s just that I share an apartment with two roommates, so if I’m singing or I’m mimicking, that kind of brings it down a little bit, makes it difficult for me." (p.377) |
| Finding 59 | Reluctance to abandon established avoidant coping strategies (U) |
| Illustration | "Especially steps 3 and 4, social contacts and friends and family, getting in contact with people doesn’t become a priority to me. What becomes a priority is shutting down my thinking, because I don’t want to think or feel any emotions." (p.378) |
| Finding 60 | Depression related lethargy [as a barrier] (C) |
| Illustration | "I'm not going to reach for my folder and, you know, look for my suicidal algorithm there, no." (p.378) |
| Finding 61 | Sometimes [consumers] are overwhelmed so quickly that they felt they had no time to act on their own behalf (U) |
| Illustration | "Sometimes I get tunnel vision and I don't get a chance to make the call but a lot of times I keep it with family so that's a good thing. Keeping with family is good" (p.378) |
| Finding 62 | When suicidal thoughts intruded on weekends or at night, and the veteran was unable to reach his/her own doctor, the plan seemed useless, especially if the doctor was perceived as the only reliable source of help (C) |
| Illustration | "The attacks happen at night so I have to call a complete stranger, the army suicide hotline, that’s a little weird. So sometimes I have to think about it before I call because I don’t feel like picking up if that’s my best option because I don’t want to talk to a complete stranger because they don’t know me" (p.378) |
| Finding 63 | Sharing it with supportive others, such as friends and family members [as a facilitator] (U) |
| Illustration | "...as one veteran noted, doing so “keeps me on my toes. And also my wife brings it up and my boys tell me how I’m doing." (p.378) |
| **Levandowski BA, Cass CM, Miller SN, Kemp JE, Conner KR. An intervention with meaning: Perceptions of safety planning among veteran health administration providers. Crisis- J Crisis Interv Suicide Prev. 2017;38(6):376–83.** | |
| Finding 64 | Systematic approach to safety planning was considered to be beneficial (U) |
| Illustration | "I’ve been a practitioner for many, many years, and if you were dealing with somebody with suicidal thoughts, you’d ask the standard questions. But suicide safety planning takes it … in a different direction with really delving into the emotions and situations, and some of those risk factors … [that] were normally in my conversations, so I think it’s an excellent addition." (p.378) |
| Finding 65 | Filling out the safety plan encompassed more than simply filling in the template (U) |
| Illustration | "I take usually about 20 to 30 minutes, because it’s not just developing the safety plan. It’s kind of talking to them and explaining why it’s useful and getting their buy-in and then helping them to complete it. So that they leave with a completed document and hopefully have bought into … the reason this document’s helpful." (p.378) |
| Finding 66 | Challenges with finding sufficient time (U) |
| Illustration | "If you’re going to expect providers who have a half an hour … to prescribe, look at lab work, follow up on discharge, … medication changes, then [meaningful safety planning] … is challenging." (p.378) |
| Finding 67 | Safety plans were updated if the patient indicated their stress levels increased, suicidal thoughts had returned or increased, or if they attempted suicide (U) |
| Illustration | "I’ll also review them anytime that there is an episode, if someone says, ‘Well, you know. Well, I thought about it.’ [My response would be] ‘So, how’d you cope with that? What did you do?’" (p.379) |
| Finding 68 | The context in which the safety plan is completed also influenced the shared experience (U) |
| Illustration | "Typically, it’s easy to do. What is sometimes difficult is [when patients] … are unable to think about anything to do … or that they don’t have anybody, whom they can talk [to] … I don’t think they are in immediate risk of actually going home and harming themselves… But at the same time, they really can’t think of things that they could do should they … have a crisis. That’s what I find most challenging." (p.379) |
| Finding 69 | Some [clinicians] also thought that safety planning served to prevent unnecessary hospitalizations (U) |
| Illustration | "I think if I didn’t have this as a tool to use, there would be a lot of liability and ethical concerns that would probably require me to hospitalize someone a lot sooner than I think is really required. I think the safety plan is a really good tool to help me help someone live independently and help them learn the skills that they need to cope with their crisis. Because this is something … that is very helpful to crisis in general … it greatly improves what we do." (p.380) |
| Finding 70 | [the SPI] is used for prompting a range of potential strategies if the veteran is having trouble identifying their own ideas (U) |
| Illustration | "I do a lot of open-ended questions to try to ensure that what they’re giving me is something from them rather than me prescribing it." (p.380) |
| Finding 71 | Personalization methods included documenting any suicide method(s) the veteran has considered and what specific steps they will take to stop themselves (U) |
| Illustration | "One veteran … was considering hanging himself and had picked out which beam in his garage he was going to hang the rope from. And he came up on his own that he had already placed a picture of his daughter on that beam to prevent him from using that." (p.380) |
| Finding 72 | Several [clinicians] noted that they did not know if safety planning was effective (U) |
| Illustration | "I think it can be helpful, but … ultimately I think that folks when they’re out there in their day-to-day world are probably not referencing that document whenever they’re in crisis." (pp.380-381) |
| Finding 73 | The safety plan supports both providers and veterans (U) |
| Illustration | "I’ve had Veterans tell me that they’ve referred to it at times and gone [to] the lower level of the safety plan, like implementing what they can do to self-soothe … that has actually helped to the point where they didn’t need to even go further or even go all the way up the chain of command to me." (p.381) |
| **Matthieu MM, Morissette SB, Clafferty S, Degutis L, Oliver CM, Adkins DA, et al. Veteran Experiences With Suicide Ideation, Suicide Attempt, and Social Support in Safety Planning Within the Department of Veterans Affairs. Mil Med. 2023 Nov 3;188(11–12):e3289–94.** | |
| Finding 74 | Identifying warning signs was the most remembered proportion (C) |
| Illustration | "identifying the signs and knowing your boundaries." (e.3292) |
| Finding 75 | Other steps veterans used included identifying warning signs, contacting family or crisis lines, and using coping strategies (U) |
| Illustration | “I've used it to identify when I'm getting into a danger zone and what I can do to help alleviate that.” (e.3292) |
| Finding 76 | The plan reminded the veteran about personal coping strategies, options for using the plan, and ways to keep their environment safe (C) |
| Illustration | “Even when I’m confused it can give me some direction." (e.3292) |
| Finding 77 | Some veterans felt the safety plan would not be useful (C) |
| Illustration | "If I really wanted to do it, I would do it and no one could, no one…I don't know. I just don't believe anybody could stop anybody who's suicidal, you know?" (e.3292) |
| Finding 78 | Subjective data [from support persons] could improve the provider’s knowledge of circumstances and triggers surrounding the patient’s suicidal ideation or behavior (C) |
| Illustration | “… at least one family member that’s up to date with all the stuff especially one that’s close.” (e.3292) |
| Finding 79 | Benefits of directly involving family members in safety plans (U) |
| Illustration | "They [family member] did something, though as far as my husband and my gun was locked up in different gun safes, and he had the keys and then my medications were locked up and, oh, I had a bunch of phone numbers" (e.3292) |
| Finding 80 | Mindfulness practices may be harder to engage in times of severe crisis or distress (C) |
| Illustration | "I’ve never utilized … the mindful techniques that I have in there, … some of the coping skills, … activities, and stuff of that nature that are written in there, but I’ve never actually pulled it out and said, ‘okay, what do I do now’ type of thing." (e.3292) |
| Finding 81 | Veterans had no desire to use their plan (C) |
| Illustration | “I mean, when you really just want to do something, I don’t know, I’m not going to go to no sheet of paper.” (e.3292) |
| Finding 82 | The safety plan should provide the veteran with options and list any scenarios and actions that should be taken while in crisis (U) |
| Illustration | “Plan out what could possibly happen, and the outcomes and you have it written down then you won’t find yourself doing something spur of the moment.” (e.3292-3293) |
| **Patel SR, Sullivan SR, Mitchell EL, Jager-Hyman S, Stanley B, Goodman M. Qualitative Study of Telehealth Delivery of Suicide-Specific Group Treatment “Project Life Force”. J Technol Behav Sci. 2023 Jan 4;8(3):272–81.** | |
| Finding 83 | Advantages of joining the group via telehealth (U) |
| Illustration | "I really like the telehealth, and that is just me personally, I like the video chat because it gives me convenience of doing it from home. I was walking down the street and you know with my head buds on and was in the group and I had to take care of some important business and I did not want to miss the group; I had to go and see my parole officer" (p.275) |
| Finding 84 | Group facilitators tried to ensure equitable participation and create space for each group member to speak even though they were not in person (U) |
| Illustration | "Very pleasant, very amicable, very sociable. I guess, inclusive, always felt like they were always rotating properly as far as getting enough feedback and, or enough involvement from each veteran, equally distributed throughout the session. So I really appreciate that about the instructors." (p.275) |
| Finding 85 | Reduced burden of making and rescheduling appointments and long waitlists (U) |
| Illustration | "With the telehealth when it comes to dealing with the group especially. I get undivided attention. Because I know that there's a guarantee that I'll have a session that Wednesday, number one. Then number two, I had a struggle with the Bronx VA of trying to not only get a psychiatrist but to get my therapy, therapy going. And it took them months. At least with this I know every Wednesday, there is group." (pp.275-277) |
| Finding 86 | Privacy concerns before joining PLF-T (U) |
| Illustration | "There were sometimes where my girlfriend was in house and certain things, I didn't want her to hear, so I had to have her go to other room and I had to, you know, be quiet or careful. So, I guess because, you know, sometimes when there's people around, you know, you don't have that privacy." (p.277) |
| Finding 87 | the [SPI] groups helped [consumers] learn to identify warning signs, and understanding the connection between their depression, PTSD and substance use disorder and suicidal thoughts, urges and plans (U) |
| Illustration | "So warning signs. If I have felt something that I don't know in the moment how I am feeling, I look at my safety plan, look on my warning signs and try to identify how I am feeling and try to do something to distract myself." (p.277) |
| Finding 88 | The ability to connect with other Veterans having similar experiences and receiving input from others helped [consumers] feel supported (U) |
| Illustration | "Well, we are Veterans and we are brothers and sisters. So, we wanna heal because you might have something that, that I have and you might have some treatment that make him feel better. We help each other out." (p.277) |
| Finding 89 | [the SPI allowed consumers to] open up and disclose their suicidal thoughts to the group or other support persons, share details about emotionally challenging periods during COVID and isolation, and ask for group input on problems that wanted help in solving (U) |
| Illustration | "I mean, let's face it, you know, bringing up a conversation about how I feel suicidal is not, is not something you would do at dinner with your friends. So just being in a group with people who were talking about it and sharing those thoughts and those experiences they were having, I mean I just opened up right away. For me it was just good right away, it was just, it was an instant connection to the telehealth group." (p.277) |
| Finding 90 | [the SPI] helped [consumers] learn how to use distraction to put time between thoughts and actions and identify the need to speak to someone when in a time of crisis (U) |
| Illustration | "So it helped prepare me a little bit, helped prepare me a little bit more. So in other words you know if, if , if 10 is the highest for the place I most don't want to be, and 1 is being in this good place, you know uh it kind of helped me to evaluate some things in a way that I can address the issue at 4, 5, or 6 opposed to waiting to get to 8 or 9 to try address it. So I guess having those rules helped me to connect the dots a little better and to come up with um a game plan on how to um deal with things, cope with things a little better." (p.278) |

U = unequivocal, C = credible; ^page numbers not available for illustrations from this article, as the article is published in an online only journal with no page numbers.
